# Highly Reversible Li‐Insertion/Extraction in Octahedral Voids of LiNi_0.5_Mn_1.5_O_4_ Cathode With Restricted SnS Anode: Critical Role of Synthetic Approach

**DOI:** 10.1002/smsc.70365

**Published:** 2026-08-19

**Authors:** Fadiya Faisal, Akshay Manohar, Shaji Jyothilakshmi, Yun‐Sung Lee, Vanchiappan Aravindan

**Affiliations:** ^1^ Department of Chemistry Indian Institute of Science Education and Research (IISER) Tirupati India; ^2^ School of Chemical Engineering Chonnam National University Gwang‐ju Republic of Korea

**Keywords:** alloy anode, electrospinning, Li‐ion battery, octahedral insertion, spinel LiNi_0.5_Mn_1.5_O_4_

## Abstract

In this work, we explore the possibility of utilizing lithium nickel manganese oxide (LiNi_0.5_Mn_1.5_O_4_ or LNMO) as a cathode material for developing a stable and safe Li‐ion battery (LIB). The full‐cell configuration is assembled by pairing LNMO with coprecipitated SnS, a high‐capacity alloy‐type anode material that surpasses conventional anode materials, such as graphite. While LNMO is typically operated in the high‐voltage region (~4.8 V vs. Li) utilizing the Ni^2+/4+^ couple associated with tetrahedral insertion, such operation often leads to severe electrolyte degradation and parasitic reactions. To overcome this, we employ a lower‐voltage (~2.8 V vs. Li) approach that utilizes the Mn^4+/3+^ redox couple corresponding to octahedral insertion, enhancing safety and stability. However, conventionally synthesized (i.e., bulk) LNMO often fails to facilitate this insertion, prompting us to adopt the electrospinning technique to produce nanostructured LNMO with improved Li‐ion kinetics and ionic transport that supports octahedral insertion. Our work also demonstrates the importance of nanostructuring, which facilitates octahedral insertion and enables us to incorporate a low‐voltage application without compromising safety. The electrospun (E‐spun) LNMO can exhibit a higher discharge capacity and superior cycling stability in both high‐ and low‐voltage regions; however, we will focus on low‐voltage applications. This hybrid configuration, integrating a fast Li‐ion diffusion spinel cathode and a high‐capacity alloy anode, enables efficient ion transport and enhanced electrode compatibility. By narrowing the voltage window to 2–3 V versus Li, the system exhibits stable cycling performance, along with improved safety, which facilitates the development of next‐generation LIBs with balanced performance and durability.

## Graphical abstract

(Sn^0^ + Li_2_S)ǁLNMO full‐cell configuration: In this configuration, we explore the feasibility of utilizing (LiNi_0.5_Mn_1.5_O_4_ or LNMO) by activating the octahedral insertion site associated with the Mn^4+/3+^ redox couple, along with voltage‐restricted SnS with reversible alloying/dealloying reaction.

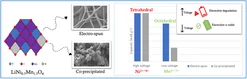

## 1 Introduction

As the world faces growing concerns over climate change and environmental degradation, reducing greenhouse gas emissions has become more urgent than ever. A major contributor to these emissions is our continued dependence on fossil fuels, which has driven global warming and other ecological challenges. To address this, there has been a global push toward cleaner, renewable energy sources, such as solar, wind, and tidal power. While these sources are sustainable and environmentally friendly, one major challenge remains in the efficient storage of these energies, as all these energy sources are intermittent. Among various energy storage solutions, Li‐ion batteries (LIBs) [1, 2] have taken the lead due to their high energy density, long cycle life, and wide applicability, from smartphones and laptops to electric vehicles. However, as our energy demands continue to rise, current LIB technologies face limitations, particularly in striking a balance between performance and safety. Although energy and power densities are crucial for advanced applications like electric vehicles, our focus shifts from increasing the voltage limits to prioritizing the safety of the devices. Rather than relying on high‐voltage systems that often compromise long‐term stability and thermal safety [3], we explore design strategies that enable reliable performance under safer, lower‐voltage conditions.

As an example of this safety‐focused approach, we utilize lithium nickel manganese oxide (LiNi_0.5_Mn_1.5_O_4_ or LNMO) in a low operating potential window. Specifically, we employ LNMO as a cathode material, as it is well known for its Co‐free composition [4] and robust three‐dimensional Li‐ion diffusion pathways [5]. While LNMO is typically preferred for its high‐voltage operation (~4.8 V vs. Li) [6, 7], which enhances energy density, this often comes at the cost of increased side reactions, thermal instability, and electrolyte degradation [3]. To avoid these issues, we intentionally cycle LNMO at a much lower potential range (2.8 V vs. Li), shifting the design paradigm toward enhanced safety and longevity. At this voltage, we minimize the risk of high‐voltage‐induced degradation while still utilizing the three‐dimensional Li‐diffusion network and octahedral voids, which support efficient Li‐transport and storage. This strategy demonstrates that with thoughtful material design and voltage control, it is possible to maintain practical performance without compromising on cell safety and structural integrity. Here, we adopt an electrospinning technique, instead of the conventional synthesis of LNMO *via* coprecipitation [8, 10], to gain better control over morphology, ion transport, and structural integrity. Electrospun (E‐spun) LNMO, with its one‐dimensional nanofibrous architecture, offers enhanced Li‐ion diffusion, increased surface area, and improved contact between the active material and electrolyte, making it suitable for both high‐ and low‐voltage operations [11]. Apart from this, nanostructuring can also enable them to accommodate stresses during Li‐insertion/extraction.

However, in our design strategy, as discussed above, we intentionally cycle the E‐spun LNMO at a lower potential range (2.8 V vs. Li), utilizing the octahedral voids within the spinel framework in a way that prioritizes safety over maximum energy output. Operating at this reduced voltage helps mitigate high‐voltage side reactions, electrolyte decomposition [5], and thermal stress, while still leveraging the structural advantages provided by the E‐spun framework. This approach can help us shift toward safer, application‐oriented battery designs, where the material’s full potential aligns with stability, durability, and practical performance needs. Building on this direction, some of our previous work showed how one‐dimensional architecture can improve the Li‐ion insertion mechanisms [9, 12]. Here, we will further explore how this nanoarchitecture not only improves the capacity retention but also aligns naturally with safer, application‐oriented configurations [13]. It also demonstrated the influence of nanostructure on reversible Li‐insertion into the void sites of LNMO [13], as E‐spun nanofibers showed a higher order of magnitude of diffusion coefficients than their bulk counterparts [13]. Despite all these advancements, most full‐cell configurations, including E‐spun LNMO as the cathode, have low‐capacity anodes like graphite [14] and LTO [15], which inherently restrict the achievable energy density. Hence, it is high time that we explore and integrate high‐capacity anodes like tin sulfide (SnS) that can complement the stable, low‐voltage operation of E‐spun LNMO.

Graphite, being the most traditional anode material, was widely used for numerous full‐cell configurations. However, its relatively low theoretical specific capacity (372 mAh g^–1^) [14, 16] is a disadvantage when it comes to the overall energy density of LIBs. This has prompted researchers to explore alternatives beyond graphite, which can store more lithium. One such material is SnS, an inorganic compound with a maximum theoretical capacity of ~1138 mAh g^–1^ (for combined conversion and alloying reaction), which is approximately three times that of graphite. SnS, being an alloying and conversion‐type anode, has less polarization and irreversibility compared to other conversion‐type anodes, making it an appealing candidate for our battery configuration. SnS also works at a relatively low redox potential, making it a great match for cathodes like LNMO. However, it comes with a challenge: SnS tends to expand significantly during charging and discharging, which can damage the electrode over time and reduce battery life. To counter this, an attempt has been made using nanostructured designs and composites to withstand volume changes and enhance the overall stability of SnS‐based anodes. In our approach, we take this step further by optimizing the system to prevent excessive volume expansion through potential control. Specifically, we restrict the electrochemical window to enable only the alloying reaction (utilizing only 4.4 moles of Li, i.e., theoretical capacity of ~782 mAh g^–1^), eliminating the conversion reaction that typically causes severe structural degradation. Restricting the potential range (5–800 mV vs. Li) maintained the structural integrity of the anode and enhanced the cycling performance.

In this work, we present a novel full‐cell architecture combining E‐spun LNMO as the cathode and coprecipitated SnS as the anode, synthesized at room temperature conditions, designed with a focus on safety, stability, and performance. By narrowing the voltage window (2–3 V vs. Li), we enhance LNMO’s structural integrity and electrochemical stability while also limiting SnS lithiation to the alloying mechanism [17]. The tailored morphologies and Li‐diffusion pathways in both electrodes promote efficient ionic transport and strong electrode compatibility. Electrochemical results demonstrate promising energy density, extended cycle life, and stable operation. These findings emphasize the importance of material and voltage design in advancing safe and high‐performance LIBs.

## 2 Results and Discussion

**FIGURE 1:**
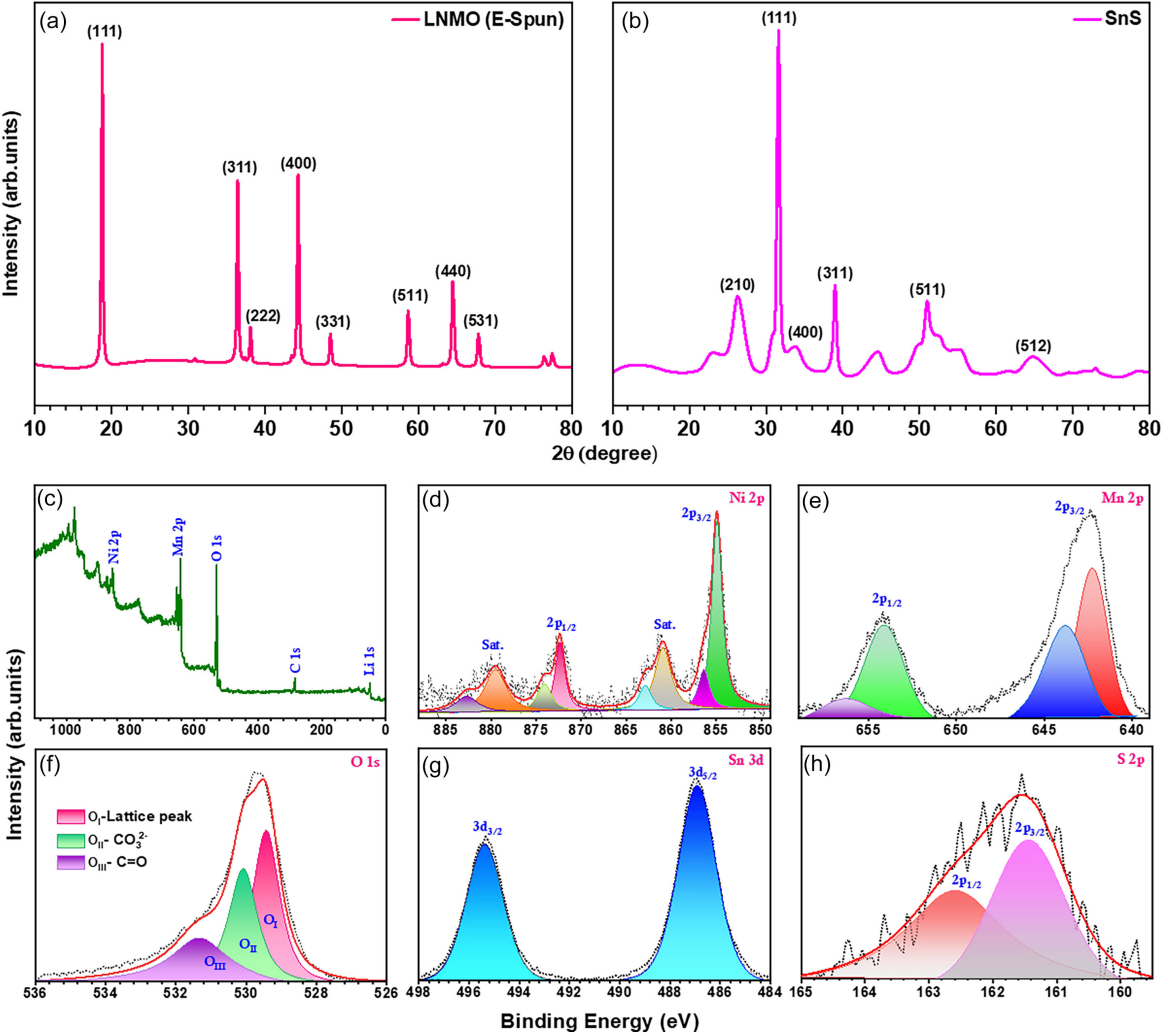
Physical characterization of E‐spun LNMO and SnS nanoparticle: (a,b) XRD pattern of LNMO and SnS, (c) XPS survey spectrum of LNMO, and (d–h) deconvoluted XPS spectrum of Ni 2p, Mn 2p, O 1s, Sn 3d, and S 2p, respectively.

The phase purity and crystallinity of the synthesized LNMO and SnS samples were examined by powder X‐ray diffraction (p‐XRD), and the results are shown in Figure 1a,b. The E‐spun LNMO sample exhibited well‐defined diffraction peaks corresponding to the formation of the cubic spinel phase, indicating its excellent crystallinity. The presence of high‐intensity reflections confirmed the formation of disordered LNMO with the *Fd3̅m* space group [18] (*DB card number: 01‐080‐5507*) and lattice parameters *a = b* = *c* = 8.16 Å. These intensity peaks can be indexed to a spinel phase, and the diffraction peaks can be matched with previous reports [13]. Compared with the bulk LNMO counterpart (Figure S1) with lattice parameters *a = b* = *c* = 8.17 Å, which was synthesized for the comparative analysis, the E‐spun sample displayed a higher degree of crystallinity [13]. The X‐ray diffraction (XRD) data of SnS are shown in Figure 1b, in which the peaks correspond to the orthorhombic crystal structure with *Pmcn* space group and lattice parameters *a =* 4.01, *b* = 4.28, and *c* = 11.21 Å (*DB card number 01‐075‐2115*). The average crystallite size of both LNMO and SnS was also calculated from the XRD using the Debye–Scherrer equation (ES_1_), which yielded values of 26.5 and 8.1 nm, respectively. Figure S2 shows the Raman spectra of E‐spun LNMO, which further confirms that spinel formation took place with a disordered *Fd3̅m* space group. The presence of a sharp peak at ~626 cm^–1^ relates to the Mn–O stretching vibration that indicates the +4 state of Mn. In addition, the peak at ~499 cm^–1^ relates to Ni–O stretching vibration and indicates the +2 state of Ni [19]. The surface chemical composition and elemental oxidation states of LNMO were examined using X‐ray photoelectron spectroscopy (XPS), as shown in Figure 1c–f. The survey spectrum (Figure 1c) confirmed the presence of Li, Ni, Mn, and O, without any noticeable impurity peaks, thereby verifying the phase purity of the material. The deconvoluted Ni 2p spectrum (Figure 1d) exhibited four main peaks at binding energies around 854.9 (Ni^2+^), 856.2 (Ni^3+^), and 872.3 (Ni^2+^), 874.1 eV (Ni^3+^), corres‐ponding to Ni 2p_3/2_ and Ni 2p_1/2_, respectively, along with characteristic satellite peaks, confirming the coexistence of Ni^2+^ and Ni^3+^ oxidation states [20]. Similarly, the Mn 2p spectrum (Figure 1e) showed peaks at ~642.25 (Mn^3+^), 643.85 (Mn^4+^), and 654.1 (Mn^3+^), and 656.1 eV (Mn^4+^), attributed to Mn 2p_3/2_ and Mn 2p_1/2_, indicating the presence of Mn^4+^ and Mn^3+^ species within the spinel lattice, confirming the octahedral insertion in LNMO [20, 21]. The O 1s spectrum (Figure 1f) displayed a dominant peak near 529.65 eV, associated with lattice oxygen (O^2–^) in the metal–oxygen framework [22]. These XPS results confirm the successful formation of a mixed‐valent spinel structure of LNMO, consistent with its expected stoichiometry and oxidation states. Similarly, the XPS analysis of the SnS sample confirmed its phase purity and chemical composition. The spectrum (Figure 1g,h) revealed the presence of only Sn and S elements, with no detectable impurity peaks. The deconvoluted Sn 3d spectrum showed two prominent peaks at binding energies of ~486.4 and 494.8 eV, corresponding to Sn 3d_3/2_ and Sn 3d_1/2_, respectively, indicating that Sn exists predominantly in the +2 oxidation state (Sn^2+^). The energy separation between these two peaks (~8.4 eV) aligns well with the reported values for SnS. Likewise, the S 2p spectrum exhibited peaks centered at 160.9 and 162.1 eV, assigned to S 2p_3/2_ and S 2p_1/2_, respectively, confirming the presence of sulfide ions (S^2–^) in the reduced state [23]. These results collectively verify that the synthesized SnS phase is pure and chemically well defined. The Brunauer–Emmett–Teller (BET) surface areas of the samples were analyzed from N_2_ adsorption/desorption studies, and their corresponding isotherm and Barrett–Joyner–Halenda pore size distribution plots are shown in Figure S3. The E‐spun LNMO and SnS nanopowders have a BET surface area of 17.9 and 44 m^2^ g^–1^ with pore volume/pore radius of 0.168 cc g^–1^/18.6 Å and 0.348 cc g^–1^/17.5 Å, respectively.

**FIGURE 2:**
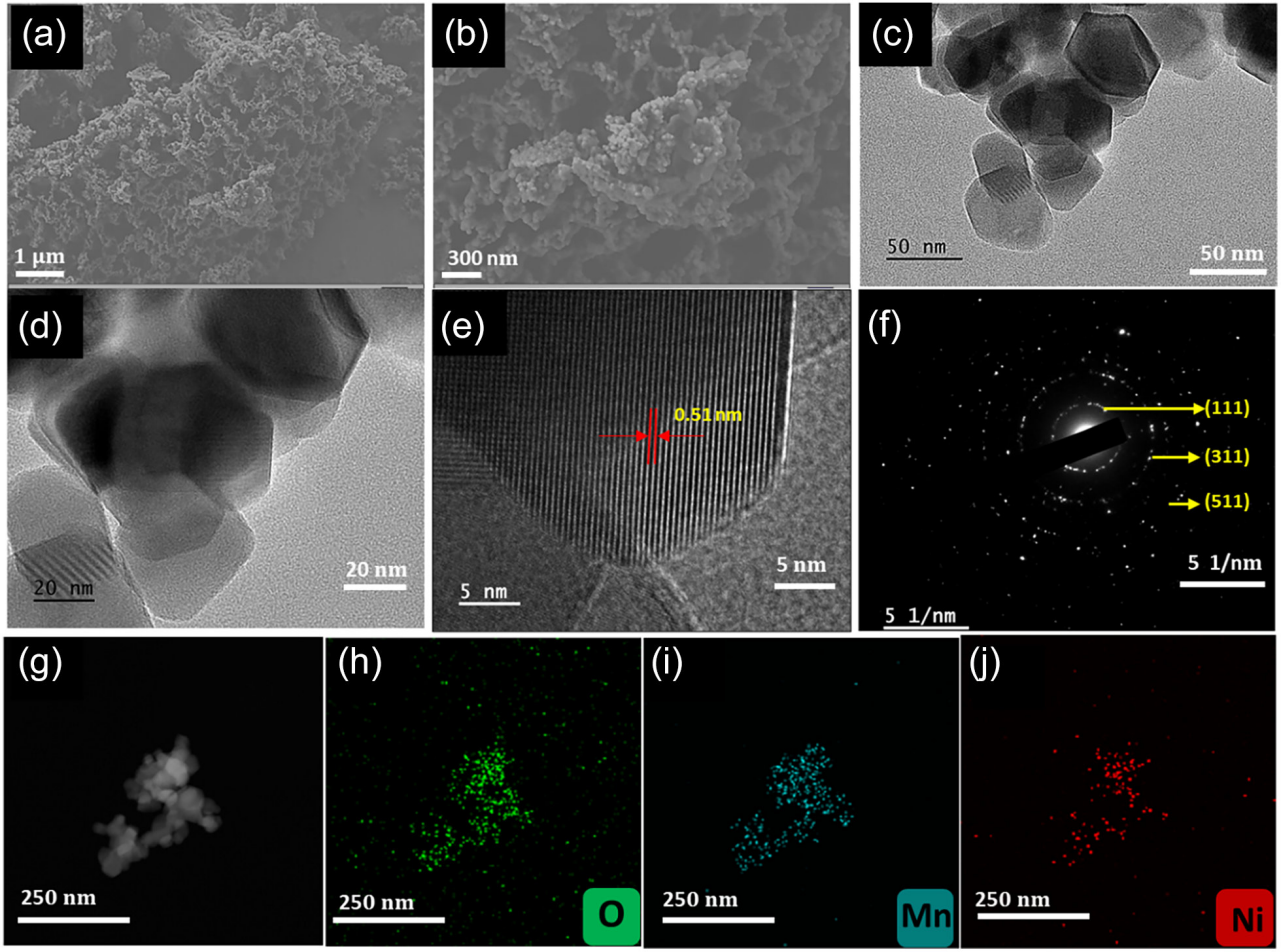
Morphological characterization of E‐spun LNMO: (a,b) FE‐SEM images, (c,d) TEM images at low magnification, (e) HR‐TEM image, (f) SAED pattern, and (g–j) elemental mapping.

**FIGURE 3:**
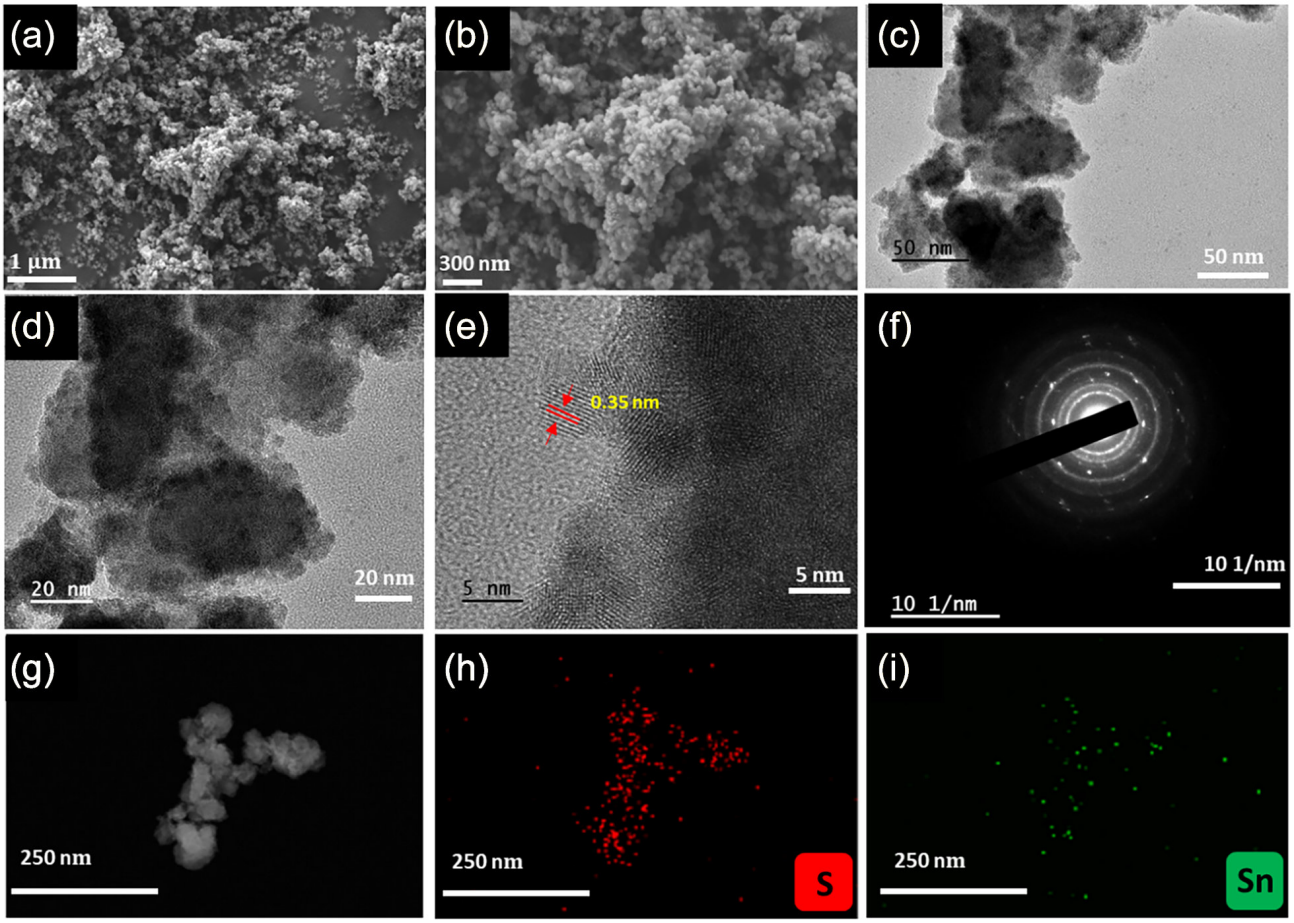
Morphological characterization of SnS: (a,b) FE‐SEM images, (c,d) TEM images at low magnification, (e) HR‐TEM image, (f) SAED pattern, and (g–i) elemental mapping.

From the field‐emission scanning electron microscope (FE‐SEM) analysis, we confirmed the fibrous morphology of the LNMO, as shown in Figure S4a–c, which was further converted into homogeneous nanoparticles after the calcination step. Post‐heat treatment at 750 °C led to the decomposition of organic moieties (such as PVP), leading to the removal of the polymer backbone, causing a conversion of fibers into polycrystalline nanoparticles Figure 2a,b [12]. These nanoparticles provide a continuous porous pathway for the easy diffusion of the Li^+^ ions. The coprecipitated LNMO, on the other hand, exhibits an agglomerated morphology with larger particles and density, as shown in Figure S4d,e. This difference in particle size has significantly impacted the electrochemical performance, which is well discussed in the upcoming sections. We were also able to synthesize the nanosized SnS at room temperature conditions, as shown in Figure 3a,b, where an agglomerated spherical morphology was observed. Moreover, the size and internal morphology of the E‐spun LNMO and SnS samples were analyzed using an high‐resolution transmission electron microscope (HR‐TEM), as shown in Figures 2c–e and 3c–e, with the interplanar distance calculated from the image being about 0.51 and 0.35 nm, respectively. The selected area electron diffraction (SAED) pattern provides the details regarding the crystallinity of the materials, where the circular spots represent the polycrystalline nature of LNMO and SnS in Figures 2f and 3f, respectively. Additionally, the elemental distribution within the LNMO samples was examined using transmission electron microscopy–energy dispersive X‐ray spectroscopy (TEM–EDS) and scanning electron microscopy–energy dispersive X‐ray spectroscopy (SEM–EDS) elemental mapping, which confirmed the uniform distribution of Ni, Mn, and O throughout the nanostructure (Figures 2g–j and S5a–c). SEM–EDS elemental mapping, for the coprecipitated sample, also showed uniform distribution of Ni, Mn, and O throughout (Figure S4f–i). Similarly, the SnS elemental mapping also detects the Sn and S elements all over the area, with no impurity elements present (Figures 3g–i and S5d–f).

### 2.1 Electrochemical Performance

The spinel LNMO features a three‐dimensional network for Li‐ion transport, enabling Li‐ion insertion in both tetrahedral (8*a*) and octahedral (16*c*) voids within the spinel lattice. The Ni^2+^/Ni^4+^ redox couple is electrochemically active in the higher‐voltage region (~3.5–5 V vs. Li) [24, 25], which facilitates Li‐ion insertion into tetrahedral sites, whereas the Mn^4+^/Mn^3+^ redox couple, which is active in the low‐voltage region (~2.2–3.2 V vs. Li), helps in the insertion of Li‐ions into the octahedral sites of LNMO [26]. The Li/LNMO stores charge by reversible redox reactions within the spinel. There are various reports on the cycling analysis of LNMO at higher voltage (3.5–5 V vs. Li) [6, 27], and it is well known that the redox activity occurs ~4.8 V versus Li (Figure S6a). The cell exhibits better specific capacity and energy density when cycled at higher voltages; however, the cycling performance would obviously be poor due to electrolyte degradation and the resulting parasitic reaction, which is one of the issues associated with the LNMO cathode. This issue can be mitigated by storing the Li‐ions in the octahedral (16*c*) voids at slightly lower voltage, which can enhance the cycling performance and safety of the device. On this note, our studies are restricted to the potential window of 2.2–3.2 V versus. Li.

**FIGURE 4:**
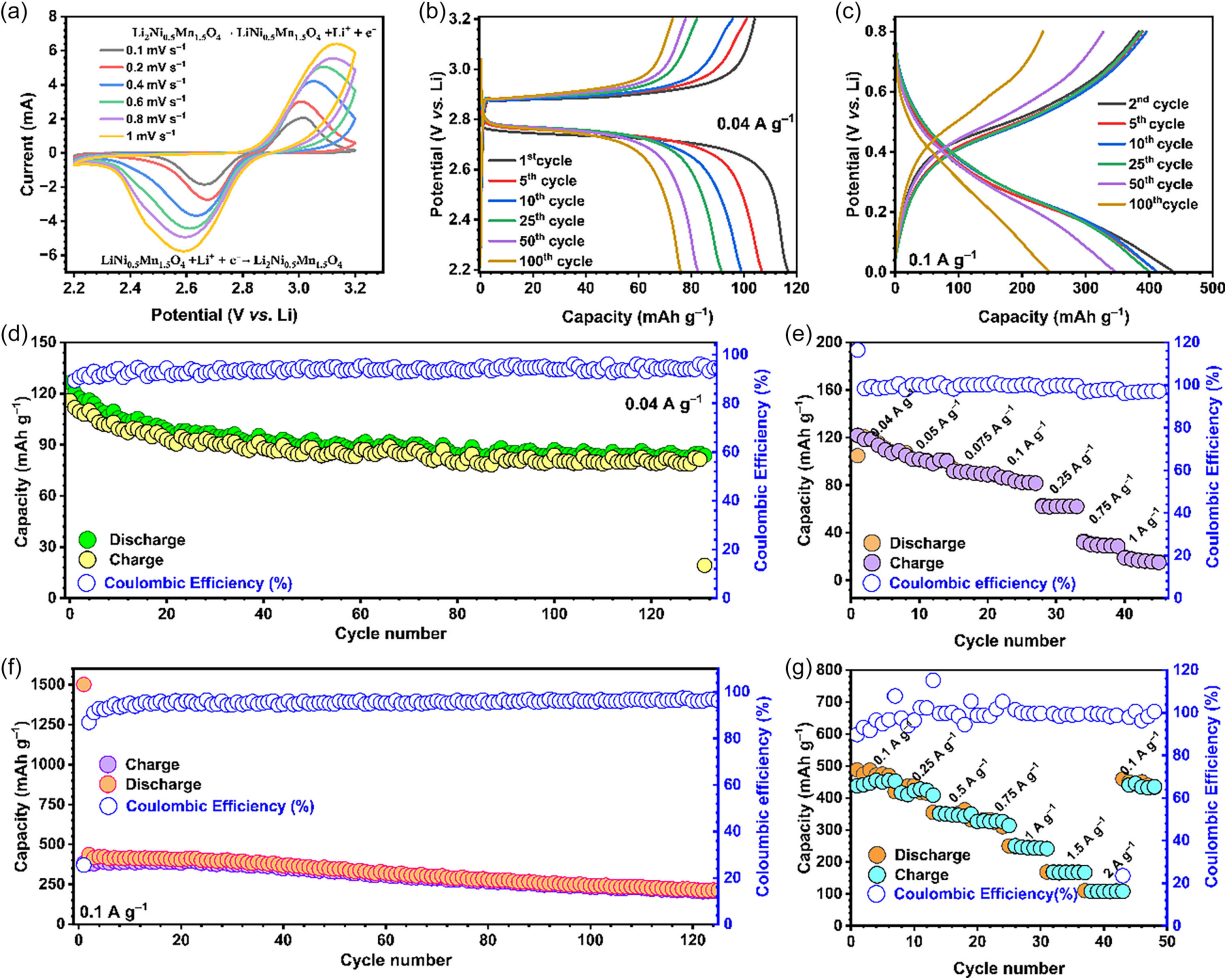
Electrochemical performance of E‐spun LNMO and SnS in half‐cell configuration: (a) CV of E‐spun LNMO at different scan rates (0.1–1 mV s^–1^), (b) GCD curves of E‐spun LNMO at a current density of 0.04 A g^–1^, (c) GCD curves of SnS at a current density of 0.1 A g^–1^, (d) long‐term cycling profile of LNMO at 0.04 A g^–1^, (e) rate performance study of Li/LNMO half‐cell at different current rates (0.04–1 A g^–1^), (f) long‐term cycling profile of SnS at 0.1 A g^–1^, and (g) rate performance profile of Li/SnS at different current rates (0.1–2 A g^–1^).

Initially, cyclic voltammetry (CV) analysis was performed for the Li/LNMO half‐cell in the lower potential window (2.2–3.2 V vs. Li). Figure 4a displays the CV curves where we could observe the reduction and oxidation peaks at 2.6 and 3 V versus. Li, respectively, which correspond to the phase transition of LiNi_0.5_Mn_1.5_O_4_ + Li^+^ + e^−^ ↔ Li_2_Ni_0.5_Mn_1.5_O_4_. This is a two‐phase reaction in which the Li‐insertion into the LNMO results in the transformation of the cubic to the tetragonal phase [9, 28], where the Li‐ions are occupied at the octahedral (16*c*) voids. As both the cubic and tetragonal structures share their faces, a mixing of 8*a* and 16*c* sites occurs [29, 30]. This phase transition is associated with Li‐insertion into the octahedral voids and the Mn^4+^ to Mn^3+^ reduction, which further induces Jahn–Teller distortion in Li_2_Ni_0.5_Mn_1.5_O_4_. The interface between the two phases formed during lithiation has poor mobility and hinders Li‐ion transport, which can subsequently reduce capacity and rate performance [13]. However, in E‐spun LNMO, this does not occur because the interphase mobility is much higher in the nanofibers, resulting in better kinetic and electrochemical performance. In addition, the fibers provide structural integrity and stability by buffering the stress caused by the distortion [13].

These CV traces are almost mirror‐like, indicating excellent reversibility on Li‐insertion/extraction [9]. Furthermore, comparing the CV traces of the E‐spun nanofibers shows a better current response at both voltages, mainly due to higher Li‐ion mobility and lower charge transfer resistance. To confirm this, we conducted Li‐ion diffusivity studies using CV. The peak current during both cathodic and anodic scans was more or less the same for the electrospun material at different voltages. The diffusion coefficients, calculated using the Randles–Sevcik equation ES_2_, for E‐spun material at high voltage is calculated to be 5.49 × 10^–10^ and 9.6 × 10^–10^ cm^2^ s^–1^ for cathodic and anodic processes, respectively, and for low voltage, it is calculated to be 6.13 × 10^–10^ and 7.7 × 10^–10^ cm^2^ s^–1^, respectively (Figure S6b,d). The diffusion‐controlled energy storage process in E‐spun LNMO is confirmed by calculating the diffusion‐controlled adjustable parameter *b*‐value in the current–voltage power‐law equation (ES_3_). The obtained *b*‐value was 0.39, which is less than 0.5 and hence implies all diffusion‐controlled processes. The CV of SnS shown in Figure S6c, on the other hand, shows two reductions and one oxidation peak. A broad reduction peak at 1.1 V versus Li is observed only in the first cycle, indicating the conversion reaction of Sn^2+^ to Sn^0^ and electrolyte decomposition, resulting in the formation of the solid electrolyte interface (SEI). The conversion reaction is associated with the huge volume variation in the electrode and could cause electrode pulverization, resulting in an increase in impedance and thereby cell failure. Hence, we have limited the conversion process only to the first cycle by limiting the oxidation potential to 0.8 V versus Li, eliminating the oxidation of Sn^0^ back to Sn^2+^. The reduction and oxidation peaks at 0.2 and 0.5 V versus Li indicate the alloying and dealloying processes, respectively, for charge storage (Equation (1)).

(1) $${\mathrm{Sn}}^{0}+x{\mathrm{Li}}^{+}+xe^{-}↔{\mathrm{Li}}_{x}\mathrm{Sn}\;\left(0\leq x\leq 4.4\right)$$

The galvanostatic charge–discharge (GCD) studies of the Li/LNMO half‐cell at a current density of 0.04 A g^–1^ exhibit decent cycling performance, with an initial specific capacity of 116 mAh g^–1^ (Figure 4b) and stable charge–discharge behavior for more than 100 cycles (Figure 4d). The half‐cell also exhibited decent cycling performance at various current densities (Figure 4e). The comparison of the half‐cell cycling performance of E‐spun with bulk LNMO gives us a clear‐cut idea of our choice of electrospinning over the coprecipitation reaction. Figure S7 illustrates that the former has three times (~120 mAh g^–1^) the specific capacity compared with the latter (~40 mAh g^–1^), while utilizing the Mn^4+^/Mn^3+^ couple. In addition, at high voltage (Figure S8), the E‐spun material also dominates the coprecipitated one, with a slightly higher specific capacity and better stability. Hence, our focus is on the former. There is a better current response for the fibers compared to the bulk, mainly due to high Li‐ion mobility, shorter Li‐ion diffusion pathways that allow faster kinetics, and the higher surface area gives better contact with the current collector [11]. The Li/SnS half‐cell also exhibited enhanced performance, with a second‐cycle discharge capacity of 436 mAh g^–1^ (Figure 4c), a coulombic efficiency of ~96%, and stable cycling performance over 100 cycles (Figure 4f). The cell also displayed high‐rate capability at different current densities and a reversibility of ~97% when finally cycled at the lower current rate (Figure 4g).

**FIGURE 5:**
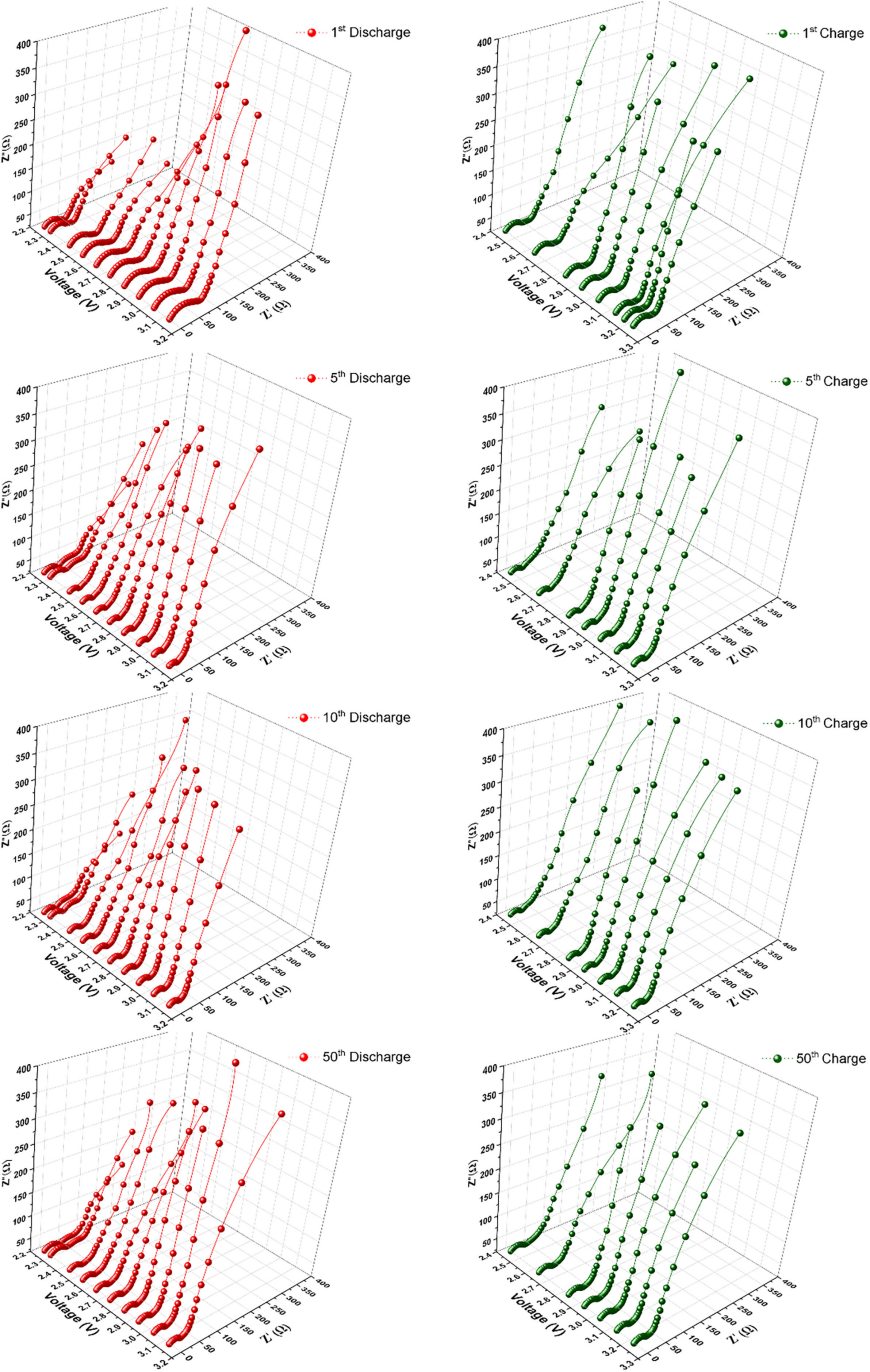
*In‐situ* EIS profile of Li/LNMO half‐cell with traces of 1^st^, 5^th^, 10^th^, and 50^th^ cycles at different potentials.

The *in*‐*situ* electrochemical impedance spectroscopy (*in‐situ* EIS) provides a powerful tool to understand how interfacial resistance evolves during cycling, giving insights into both Li‐ion transport kinetics and electrode–electrolyte stability. For the LNMO cathode, when cycled in the low‐voltage window, which corresponds to octahedral insertion governed by the Mn^4+^/Mn^3+^ redox couple the in situ EIS profiles recorded at 1^st^, 5^th^, 10^th^, and 50^th^ cycles (Figure 5) showed pronounced charge transfer resistance (*R*_CT_) in the initial discharges, reflecting the activation of the LNMO surface and the initial insertion of Li^+^ ions in the octahedral framework. In the subsequent cycles, we can see a progressive decrease and the stabilization of *R*_CT_ in both discharge (Li‐insertion) and charge (Li‐extraction) states. Lower *R*_CT_ values indicate that the LNMO nanofiber has become increasingly electrochemically accessible, enabling faster Li‐ion transport. The declining values also indicate the efficient and reversible Li‐ion insertion from the same sites. This behavior confirms that the octahedral site insertion has become more kinetically favorable with continued cycling. A similar kinetic insight is obtained for the alloy‐type anode SnS through *in‐situ* EIS, which can give insights about SEI layer formation. The SEI layer formation in SnS irreversibly consumes Li‐ions during the first discharge and results in significant irreversibility upon anodic scanning. Unlike LNMO, a uniform and stable SEI layer formation is necessary for anodes, particularly for alloy‐type anodes, because nonuniformity may result in electrolyte–electrode interaction and further degradation, leading to thickening and non‐uniform SEI layer development, resulting in irreversible Li loss. This can be severe in alloy‐type anodes, as they undergo significant volume expansion/contraction, creating stress during cycling, and may lead to degradation of the SEI layer. Hence, robust and uniform SEI is important in such anodes. An in situ EIS study was conducted for the Li/SnS half‐cell, with data recorded for the 1^st^, 5^th^, 10^th^, and 50^th^ cycles (Figure S9) to analyze the stability and influence of the SEI on electrochemical performance. We observed a higher charge transfer resistance (*R*_CT_) during the first discharge, primarily due to the formation of the SEI layer. In the initial discharge, the SEI is just forming, and the pathway across is not established. This can cause sluggish kinetics. However, in the subsequent cycles, the electrode is much more activated with the formation of stable SEI, higher surface contact, and better conductivity. Hence, we could observe a lower *R*_CT_ value in the cycle following the initial discharge.

Going deep into each potential, we could observe that at the lower cutoff potential of 5 mV versus Li, the *R*_CT_ values are found to be higher, irrespective of the charge or discharge process, due to the growth/formation/reformation of the SEI layer. When the cell potential approaches the upper cutoff potential of 0.8 V versus Li, a dramatic reduction in the *R*_CT_ is observed, which is associated with the stabilization of SEI. In addition, the different *R*_CT_ values at different potentials can be attributed to the distinct phases at different potentials. The lithiated phase (Li_*x*_Sn) is found to be less conductive than the delithiated phase (Sn^0^), which might be one of the reasons for such a trend, besides the Li_2_S matrix. This reformation and stabilization of the SEI layer are continuously seen for almost all cycles. The in situ impedance is direct evidence of how SEI formation and stabilization take place during the charge–discharge process. After the first charge–discharge cycle, the *R*_CT_ value decreased to a lower value at 0.8 V versus Li, and remained nearly constant up to the 50^th^ cycle. This observation can be attributed to the formation of a stable and uniform SEI layer during the initial cycles, which minimized side reactions and facilitated smooth Li‐ion transport, thereby enhancing the electrochemical performance of the Li/SnS half‐cell.

### 2.2 Post‐mortem Studies

**FIGURE 6:**
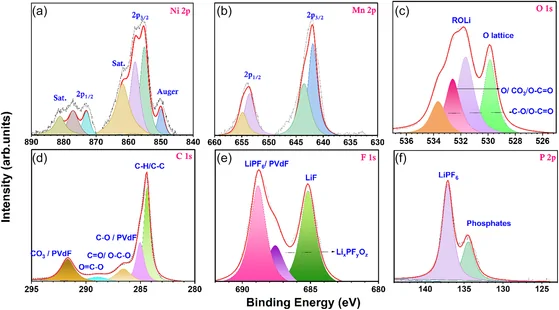
XPS spectrum of cycled LNMO electrode: deconvoluted XPS spectrum of (a) Ni 2p, (b) Mn 2p, (c) O 1s, (d) C 1s, (e) F 1s, and (f) P 2p.

The structural stability of the material was confirmed by *ex‐situ* XRD analysis of the cycled LNMO electrode (Figure S10), in which no changes in the peaks were observed, indicating that the structure remained intact even after continuous Li‐insertion/extraction and the associated Jahn–Teller distortion. The post‐cycling microscopic and elemental analysis was performed for the electrodes, and their results are shown in Figures 6, S11 and S12. There were not much difference observed in the SEM images of the LNMO electrode. We could see the uniform distribution of the nanopores/Li‐ion diffusion channels from Figure S11a–c. The post~mortem images of the SnS, on the other hand, display a completely different morphology because of the continuous alloying/dealloying process that occurred during cycling (Figure S12a,b). Figure S11d–i gives us information about the elements detected in the cycled LNMO electrode. It shows the presence of additional elements like carbon, fluorine, and phosphorus, which are added from the conductive carbon and electrolyte used during cell fabrication. Similar analyses were performed for the SnS samples, Figure S12c–h, revealing well‐dispersed nanosized particles with homogeneous distribution of Sn and S. However, other elements, such as carbon, fluorine, and phosphorus, were also present.

In addition, post‐cycling XPS analysis was also performed to study the surface of the LNMO electrode after long cycling. Elements, such as Ni, Mn, O, C, F, and P, were detected, and the deconvoluted peaks in Figure 6 provided a clear picture of the components. The Ni 2p spectrum (Figure 6a) showed four main peaks at 855.2 (Ni 2p_3/2_), 873.2 (Ni 2p_1/2_), and 857.8 (Ni 2p_3/2_), 877.2 (Ni 2p_1/2_), corresponding to Ni^2+^ and Ni^3+^, respectively. The presence of satellite peaks in the ~860 and 880 eV region confirms the coexistence of Ni^2+^ and Ni^3+^ oxidation states even after cyclings [31]. In addition, a low‐binding‐energy peak at ~850 eV is observed, attributed to an Auger transition. The Mn 2p spectrum (Figure 6b) displays peaks at ~642 (Mn 2p_3/2_), 653.7 (Mn 2p_1/2_), and 643.4 (Mn 2p_3/2_), 654.9 eV (Mn 2p_1/2_), attributed to the presence of Mn^4+^ and Mn^3+^ species and their coexistence [32], similar to pre‐cycling, and is evidence of the octahedral insertion as mentioned above. The O 1s spectrum (Figure 6c) was deconvoluted into multiple peaks. The peak positioned at ~529.6 eV is assigned to lattice oxygen in the spinel framework, while the higher‐binding energy components at ~531–533 eV are attributed to other oxygen species, including organic species that might have originated due to electrolyte decomposition [33]. The C 1s spectrum Figure 6d has several other contributors, with a dominant peak at ~284.8 eV corresponding to C─C/C─H bonds, along with additional peaks observed at ~286.2 (C–O), ~287.8 (C = O), and ~289.3 eV (O–C═O) [33]. These peaks can indicate the formation of organic components from electrolyte degradation and PVdF binder decomposition. The F 1s spectrum (Figure 6e) shows a strong peak at ~685 eV, characteristic of LiF, which arises from LiPF_6_ decomposition and can form even under low‐voltage cycling conditions [34]. In addition, contributions from PVdF‐derived fluorinated species were observed and can indicate electrolyte salt decomposition during cycling. Finally, the P 2p spectrum Figure 6f shows a dominant peak near  ~134.5 eV, attributed to LiPF_6_‐derived phosphate species [34], along with a low‐intensity peak which can be attributed to inorganic phosphate compounds [33].

### 2.3 (Sn^0^ + Li_2_S)ǁLNMO‐Based Li‐ion Battery Performance

We fabricated the (Sn^0^ + Li_2_S)ǁLNMO‐based LIB by assembling the two electrodes by following a simple mass balancing equation (ES_4_) and operated in the potential window of 2–3 V. The optimized mass ratio of anode to cathode was 1:4.1. As discussed in the experimental section, the working electrodes were fabricated and assembled into coin cells under inert conditions. The cells were then cycled at a current density estimated based on the mass loading and capacity of the SnS anode. The (Sn^0^ + Li_2_S)ǁLNMO‐based LIB is fabricated in such a way that, initially, the Li/SnS half‐cell is subjected to GCD analysis for three cycles and then terminated at the discharged state to obtain the lithiated phase (Li_*x*_Sn + Li_2_S). After the prelithiation step, the half‐cell is dismantled and paired with the mass‐balanced LNMO electrode to fabricate (Sn^0^ + Li_2_S)ǁLNMO full cell.

**FIGURE 7:**
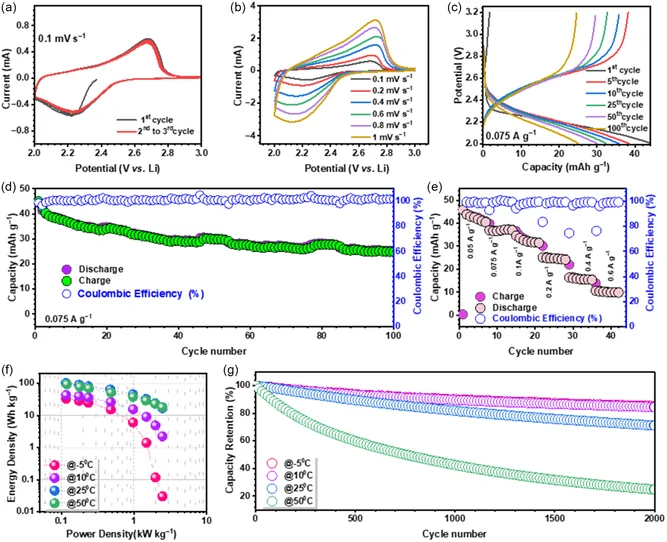
Electrochemical performance of (Sn^0^ + Li_2_S)ǁLNMO‐based LIB: (a) Cyclic voltammogram at a scan rate of 0.1 mV s^–1^, (b) CV at different scan rates (0.1 to 1 mV s^–1^), (c) GCD curves at a current density of 0.075 A g^–1^, (d) long‐term cycling at 0.075 A g^–1^, (e) rate performance study of full cell at different current rates (0.05 to 0.6 A g^–1^), (f) Ragone plot comparing energy and power density values at different temperatures (–5, 10, 25, and 50 °C), and (g) long‐term cycling profile at a current density of 0.075 A g^–1^ at different temperatures (–5, 10, 25, and 50 °C).

The CV curves recorded at a scan rate of 0.1 mV s^–1^ are shown in Figure 7a, showing distinct and stable redox peaks, indicating high reversibility. The reduction peak at 2.2 V represents the dealloying process, and simultaneous insertion of Li‐ions into the LNMO octahedral (16*c*) sites, and corresponding oxidation peaks are visible at 2.6 V, displaying the reverse reaction during the charging process. The CV curves at different scan rates (0.1 to 1 mV s^–1^) (Figure 7b) show the reversibility of the LIB with a negligible shift in the peak position. The GCD profile of our LIB was decent, with a reasonable capacity retention and Coulombic efficiency. The cell displayed an initial discharge capacity of ~45 mAh g^–1^ with a coulombic efficiency of ~99% (Figure 7c). The cell cycled at 0.075 A g^–1^ exhibited a capacity retention of 67% after 50 cycles of charge–discharge with a coulombic efficiency of >99% (Figure 7d). The cycling was also tested at different current densities from 0.05 to 0.6 A g^–1^, in which the results are shown in Figure 7e. The specific capacity appears to decrease as the current rate increases, due to insufficient time/limited kinetics for the complete reaction, as is generally the case. However, it exhibits reasonable performance at different current rates. Additionally, the rate performance study was conducted at various temperatures, ranging from the lower temperature of –5 °C to the higher temperature of 50 °C. The adaptability of our LIB to different climatic conditions was tested here, in which the cell exhibited better performance at all temperatures. The energy/power density was then calculated with the help of the Ragone plot (Figure 7f), and we could find that the best performance was exhibited at the room temperature conditions (25 °C). The LIB exhibited a maximum energy density of ~102 Wh kg^–1^ and power density of ~2.5 kW kg^–1^. These values are calculated based on the total mass of the active material in the electrode (2.57 + 10.74 mg) and using the ES_4_. With experimental capacities *C*_1_ = 460 mAh g^–1^ for the anode and *C*_2_ = 110 mAh g^–1^ for the cathode, the resulting N/P ratio is ~1. This near‐unity N/P ratio reflects a well‐balanced cell configuration, enabling optimal capacity matching between electrodes and can contribute to stable electrochemical performance.

Comparing different temperatures, the lowest temperature seems to have poor performance due to higher impedance caused by the freezing of the electrolyte. This has led to the reduction of the activity of the electrode materials, leading to the inability to extract the maximum capacity out of them. On the other hand, at the highest temperature, the activity increases to an extent, promoting unwanted side reactions that lead to degradation of the cycling performance of the LIB. This is clearly evident from the long‐term cycling analysis at 50 °C (Figure 7g), where we could observe a huge decrease in capacity as the cycle progresses. However, the cycling performance at other temperatures is far better, where we could obtain more than 70% of the capacity retention even after 2000 cycles.

Our work, with SnS as the anode and LNMO as the cathode, is a promising and interesting combination in LIB chemistry. The low‐voltage anode with high capacity and a stable cathode with high‐rate capability are notable pairs for a high‐energy, high‐power, and long‐lasting cycling LIB. The low‐voltage operation of the devices brings long‐term cycling with high‐capacity retention and coulombic efficiency. Overall, this configuration is much better than conventional rechargeable systems, including Ni‐Cd and Pb‐acid batteries, with no memory effect, lower self‐discharge rates, and greater safety, as it avoids toxic metals like lead and cadmium. Further modifications and scientific advancements are underway to enhance the electrochemical performance of the device for commercial market competition.

## 3 Conclusion

This work presents a safety‐oriented design strategy for advancing LIBs that achieve high performance without relying on high‐voltage operation. By combining nanostructural engineering with voltage limitation, we demonstrate how the electrochemical potential of LNMO can be effectively harnessed within a safer, low‐voltage window (2.2–3.2 V vs. Li). The unique nanofibrous morphology of E‐spun LNMO promotes efficient Li‐ion transport and structural stability, enabling octahedral‐site insertion while minimizing the risks of electrolyte degradation and thermal instability typically associated with high‐voltage cycling. Paired with a high‐capacity SnS alloy anode, the (Sn^0^ + Li_2_S)ǁLNMO full‐cell configuration delivers excellent energy and power performance, enhanced rate capability, and stable operation across a wide temperature range (–10 to 50 °C). The robust SEI formation and consistent interfacial behavior contribute to long‐term cycling stability and high reversibility. Overall, this study underscores the importance of coupling voltage optimization with nanoscale material design to overcome the conventional trade‐off between safety and energy density in LIBs. The (Sn^0^ + Li_2_S)ǁLNMO system thus offers a viable pathway toward next‐generation lithium‐ion batteries that combine intrinsic safety, durability, and efficiency, paving the way for scalable and reliable energy storage technologies.

## 4 Experimental Section

### 4.1 Synthesis of LNMO Nanofibers

**SCHEME 1:**
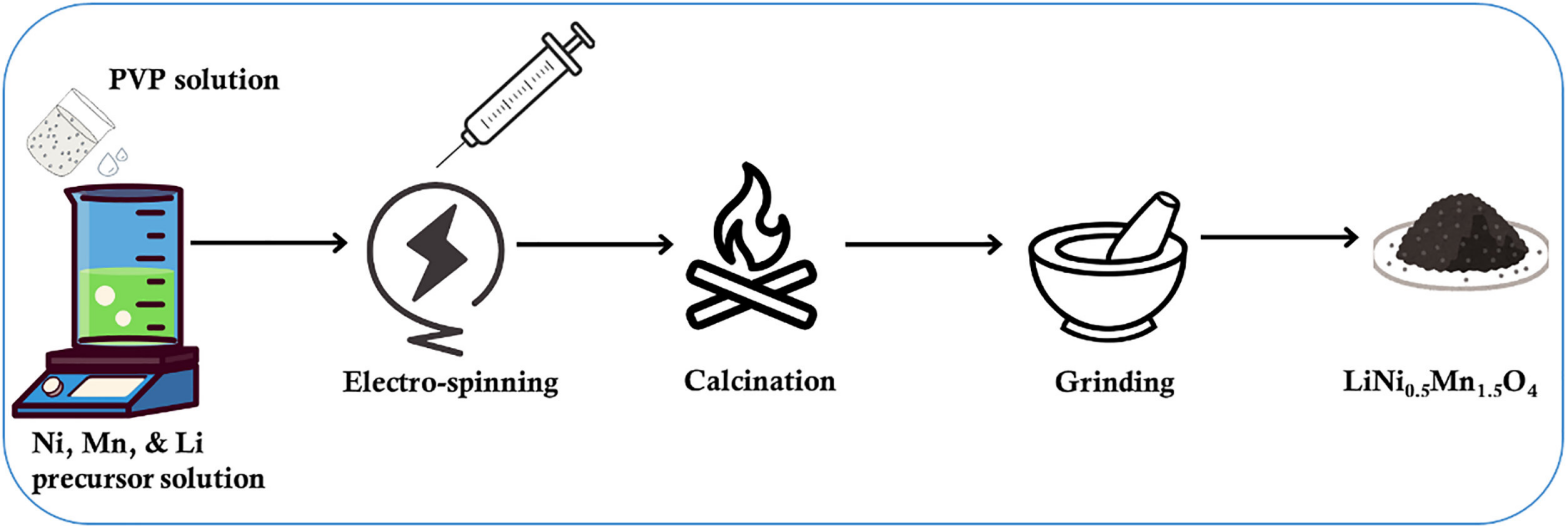
A schematic illustration of the synthesis of E‐spun LNMO.

Spinel LNMO nanofibers were synthesized via an electrospinning technique using Holmarc’s (HO‐NFES‐040D model) electrospinning unit. The synthesis protocol was adapted from a previously reported method [12, 13]. Concisely stoichiometric amount of lithium acetate (C_2_H_3_LiO_2_, 99.95%, Sigma‐Aldrich), nickel nitrate hexahydrate (Ni(NO_3_)_2_·6H_2_O, 99%, Sigma‐Aldrich), and manganese acetate tetrahydrate ((CH_3_COO)_2_Mn·4H_2_O, 99%, Sigma‐Aldrich) were dissolved in 14 mL of methanol (Sigma‐Aldrich, 99.9%) along with 0.3 mL of acetic acid (Sigma‐Aldrich) to form a precursor solution. Separately, 2.5 g of polyvinylpyrrolidone (PVP, Mw = 3,60,000 g mol^–1^, Sigma‐Aldrich) was dissolved in 7 mL of ethanol (Sigma‐Aldrich) to obtain a polymer solution. The two solutions were mixed and stirred overnight to obtain a homogeneous spinning solution with a weight percentage of ~11.7. This homogeneous solution was then loaded into a 10‐mL syringe fitted with a needle of an inner diameter of 0.83 mm and subjected to electrospinning. The following parameters were fixed to obtain LNMO nanofibers: flow rate, 15–20 mL h^–1^; applied voltage, 14 kV between the tip of the needle and a grounded Al‐foil; and distance between the tip of the needle and the current collector, 15 cm. After electrospinning, the spun fibers were preheated in a box furnace at 200 °C for 1 h at a ramp rate of 2 °C min^–1^ to remove the organic moieties. The preheated fibers were then calcined in two steps: first at 600 °C for 1 h, and then at 750 °C for 6 h, both with a ramp rate of 2 °C min^–1^, in a tube furnace to obtain the crystalline spinel phase of LNMO. The synthesis procedure is shown in Scheme 1.

### 4.2 Synthesis of SnS Nanopowder

**SCHEME 2:**
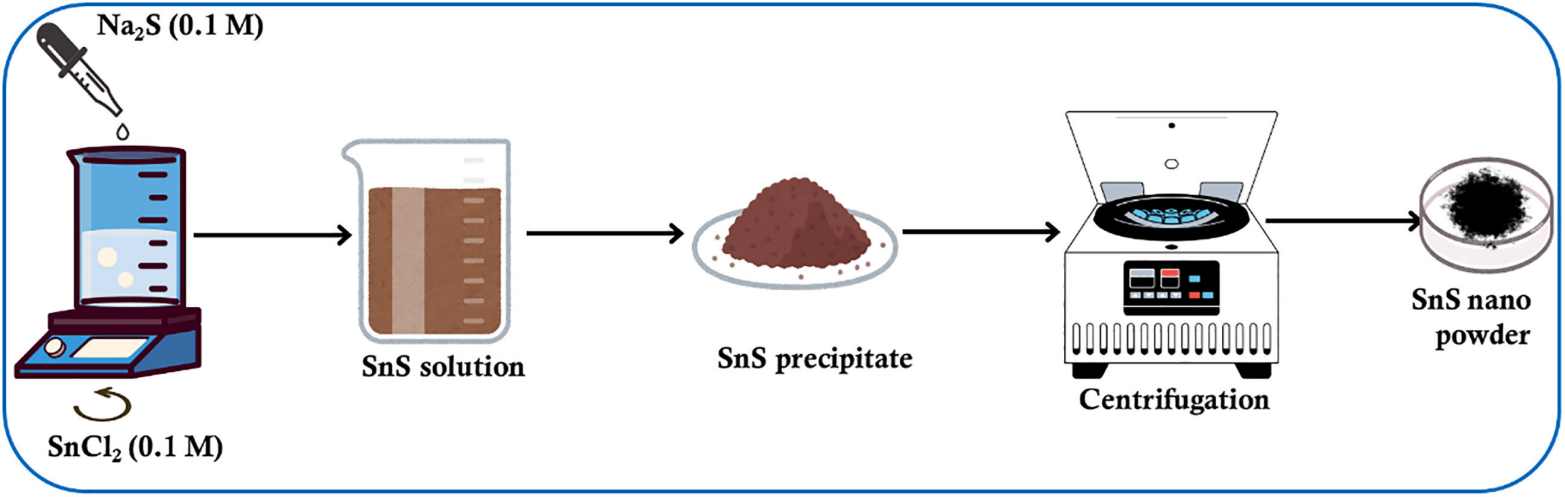
A schematic illustration of the synthesis of nano‐SnS.

We followed a similar procedure reported by Akshay et al. [23] for the synthesis of the SnS nanoparticles. It was synthesized using a room temperature coprecipitation method. 0.1 M aqueous solution of sodium sulfide (Na_2_S, 99%, Sigma‐Aldrich) was added dropwise into a 0.1 M aqueous tin (II) chloride (SnCl_2_, 99%, Sigma‐Aldrich) solution; 10 ml of HCl was also added to facilitate dissolution. A brown precipitate formed was collected by centrifugation and washed with deionized water and ethanol to remove residual NaCl impurity. The product was then dried overnight at 65 °C to yield SnS nanopowder. The synthesis procedure is shown in Scheme 2.

### 4.3 Material Characterization

The crystalline phase and purity of the synthesized materials were examined using p‐XRD (Rigaku D/teX Ultra 250 diffractometer; 40 kV, 200 mA, *λ* = 1.5406 Å with Cu K_α_ radiation). The Raman spectroscopic study for E‐spun LNMO was conducted using Lab RAM HR800 UV‐Raman microscope (Horiba Jobin‐Yvon, France) with a 532‐nm diode as the excitation source. The BET surface area and porosity of E‐spun LNMO and SnS were obtained from an automated gas sorption analyzer (Autosorb IQ‐XR–XR‐XR, 3 stat., Viton). The morphology and internal features of the samples were analyzed using a FE‐SEM (S‐4700, Hitachi, Japan) and a HR‐TEM (TECNAI, Philips, the Netherlands, 200 kV), respectively. SEM imaging was used to observe the particle shape, size, and distribution, while TEM provided high‐resolution insights into the internal structure and lattice fringes, confirming crystallinity and nanoscale features. Further analysis of the elemental composition, chemical bonding, and surface elements was performed using XPS (Multilab 2000, UK; monochromatic Al K_α_ radiation h*ν* = 1486.6 eV) and energy‐dispersive X‐ray spectra (EDS).

### 4.4 Electrochemical Analysis

Electrochemical measurements were conducted using CR2016‐type coin cells assembled in an Ar‐filled glove box (MBraun, Germany) with oxygen and moisture levels maintained below 0.1 ppm. Both half‐cell and full‐cell configurations were evaluated to study the electrochemical performance of the synthesized electrodes. For the LNMO half‐cell, the electrode was prepared using a mixture of active material (E‐spun LNMO), conductive carbon (acetylene black, AB), and binder (teflonized acetylene black, TAB‐2) in a 10:2:2 weight ratio, which was mixed in a mortar pestle with ethanol and made into a freestanding film. The resulting thin film was then manually pressed onto a 14‐mm stainless steel mesh current collector (Goodfellow, UK). These electrodes were dried overnight at 75 °C in a vacuum oven prior to use. Similar half‐cells were prepared by replacing the active material with bulk‐LNMO to conduct a comparative study on the electrochemical behavior of both morphologies. For the SnS half‐cell, the working electrode was fabricated using the conventional slurry‐casting method. The electrode slurry consisted of 80% active material (SnS), 10% conductive carbon (Super P), and 10% binder (polyvinylidene fluoride, PVdF) in N‐methyl‐2‐pyrrolidone (NMP) solvent, forming a uniform slurry. This slurry was then coated onto a Cu‐foil current collector using a doctor blade apparatus, followed by complete drying at 65 °C overnight. The coated foil was then cut into 12‐mm‐diameter electrodes (the active mass loading between 2 and 4 mg was noted), kept at 75 °C in a vacuum oven for a minimum of 4 h, before the insertion into the glove box and cell assembly.

In both half‐cells, metallic Li‐foil served as the counter/reference electrode, and a glass microfiber separator (Whatman, Cat No. 1825‐047, UK) was used to prevent electrode shorting. The electrolyte comprised 1 M LiPF_6_ dissolved in a 1:1 (v/v) mixture of ethylene carbonate (EC) and dimethyl carbonate (DMC) (Tomiyama, Japan), and a fixed volume of 100 µL electrolyte was used during fabrication of both half‐cells and full cells. Prior to the balanced full‐cell fabrication, the SnS half‐cells were subjected to three complete charge–discharge cycles, and the cells were then decrimped to carefully recover the electrodes in their discharged (lithiated, Li_*x*_Sn) state. For assembling the full cell, the mass loading of LNMO was then calculated based on the capacity and active mass of the prelithiated SnS anode, ensuring proper mass balancing between the two electrodes. In the context of full‐cell assembly, mass balancing refers to the balancing of charge between the cathode and anode, where the capacities of both electrodes are matched to ensure that neither electrode is significantly oversized. GCD and in situ EIS studies were performed using a BioLogic BCS‐805 battery tester (France). CV studies were conducted using a 1470E Solartron (multichannel potentiostat). Further temperature‐dependent studies were conducted in a programmable environmental chamber (Espec, Japan) to evaluate thermal stability and performance under varying operational conditions.

## Funding

S.J. thanks the Prime Minister’s Research Fellowship (0902009) from Govt. of India for financial support. V.A. acknowledges financial support from the Department of Science and Technology, Ministry of Science and Technology, India (Grant DST/NM/TUE/EE‐03/2019‐1G‐IISERTp). Y.S.L. acknowledges the financial support from the National Research Foundation of Korea (NRF) grant funded by the Korean government (Ministry of Science, ICT&Future Planning) (No. RS‐2023‐00208361).

## Conflicts of Interest

The authors declare no conflicts of interest.

## Supporting information

The author have cited additional references within the Supporting Information [35].

## Data Availability

The data that support the findings of this study are available in the Supporting Information of this article.
