# Supplementary material for: Highly Reversible Li‐Insertion/Extraction in Octahedral Voids of LiNi0.5Mn1.5O4 Cathode With Restricted SnS Anode: Critical Role of Synthetic Approach
Source: Small Sci. 2026 Aug 19;6(8):e70365. doi: 10.1002/smsc.70365 (PMC13489502; doi:10.1002/smsc.70365)
Supplement: Supplementary file 1 [file SMSC-6-e70365-s001.pdf]

Supplementary Information

Experimental Section

Synthesis of LNMO via Co-precipitation: We followed a similar procedure reported by *Sharma et.al* [43] for the synthesis of co-precipitated bulk LNMO. The co-precipitation technique, followed by solid-state reaction, was employed for the synthesis. Initially, aqueous precursor solution was prepared by dissolving 0.05 M nickel(II) acetate tetrahydrate ($Ni(CH_3COO)_2 \cdot 4\ H_2O$) and 0.15 M manganese(II) acetate tetrahydrate ($(Mn(CH_3COO)_2 \cdot 4\ H_2O$ (both ≥99.0% purity from Sigma-Aldrich) in deionized water. Separately, an aqueous solution of 0.1 M sodium carbonate decahydrate ($Na_2CO_3 \cdot 10H_2O$, ≥99.0%, Sigma-Aldrich) was prepared. The carbonate solution was added dropwise to the precursor solution using a burette at a controlled rate as a precipitating agent over a period of 6 h while maintaining the temperature at 60 ^{0}C. Slower addition ensured smaller and finer particles. After complete precipitation, the resulting precipitate was centrifuged and repeatedly washed with distilled water to eliminate any water-soluble impurities. Subsequently, the purified precipitate was dried at 60 °C for 24 h in a hot air oven to remove residual moisture. The dried precipitate was then hand-ground with a stoichiometric amount of lithium carbonate (Li_2CO_3, 99%, Sigma-Aldrich) to introduce the lithium source. The obtained mixture was annealed in air at 850 °C for 12 h. The cooled powder was then hand-ground and cold-pressed into pellets. The pellets were then calcined again at 700 °C for 24 h. We found that the **two-step annealing procedure, combined with pelletization before heat treatment, significantly improved the phase purity and crystallinity of LNMO. Pelletisation** ensured better particle–particle contact, thereby reducing inhomogeneities and promoting uniform diffusion of Li, Ni, and Mn ions during thermal treatment.

Equation ES1: The Debye Scherrer equation for calculating the crystallite size of the active material is given below:

$$\boldsymbol{D = \frac{K\lambda}{\beta \cos\theta}}$$

D is crystallite size (nm)

K is 0.9 (Scherrer constant)

λ is 0.15406 nm (wavelength of the X-ray sources)

β is FWHM (radians)

θ is peak position (radians)

Equation ES2: The Randles-Sevcik equation for calculating the peak current is given below:

$$I_p = (2.69 * 10^5)\ A\ n^{3/2} D^{1/2}_{Li} \nu^{1/2} C^0_{Li}$$

I_p is the peak current density (mA cm^{-2})

n is the number of electrons per reaction species

D_{Li} is the Li-diffusion coefficient ($cm^2\ s^{-1}$)

ν is the scan rate (mV s^{-1})

C^0_{Li} is the bulk concentration of lithium (0.0244 mol cm^{-3} calculated from the theoretical density of spinel)

Equation ES3: The current-voltage power-law equation. The relation of peak current (i) on scan rate (v) is given below:

$$i = av^b$$

Equation ES4: The equation for balancing the mass/charge between the two electrodes is given below:

$$m_1C_1 = m_2C_2$$

Were,

m_1 is the mass of active material in the anode

C_1 is the capacity of the anode

m_2 is the mass of the active material in the cathode

C_2 is the capacity of the cathode

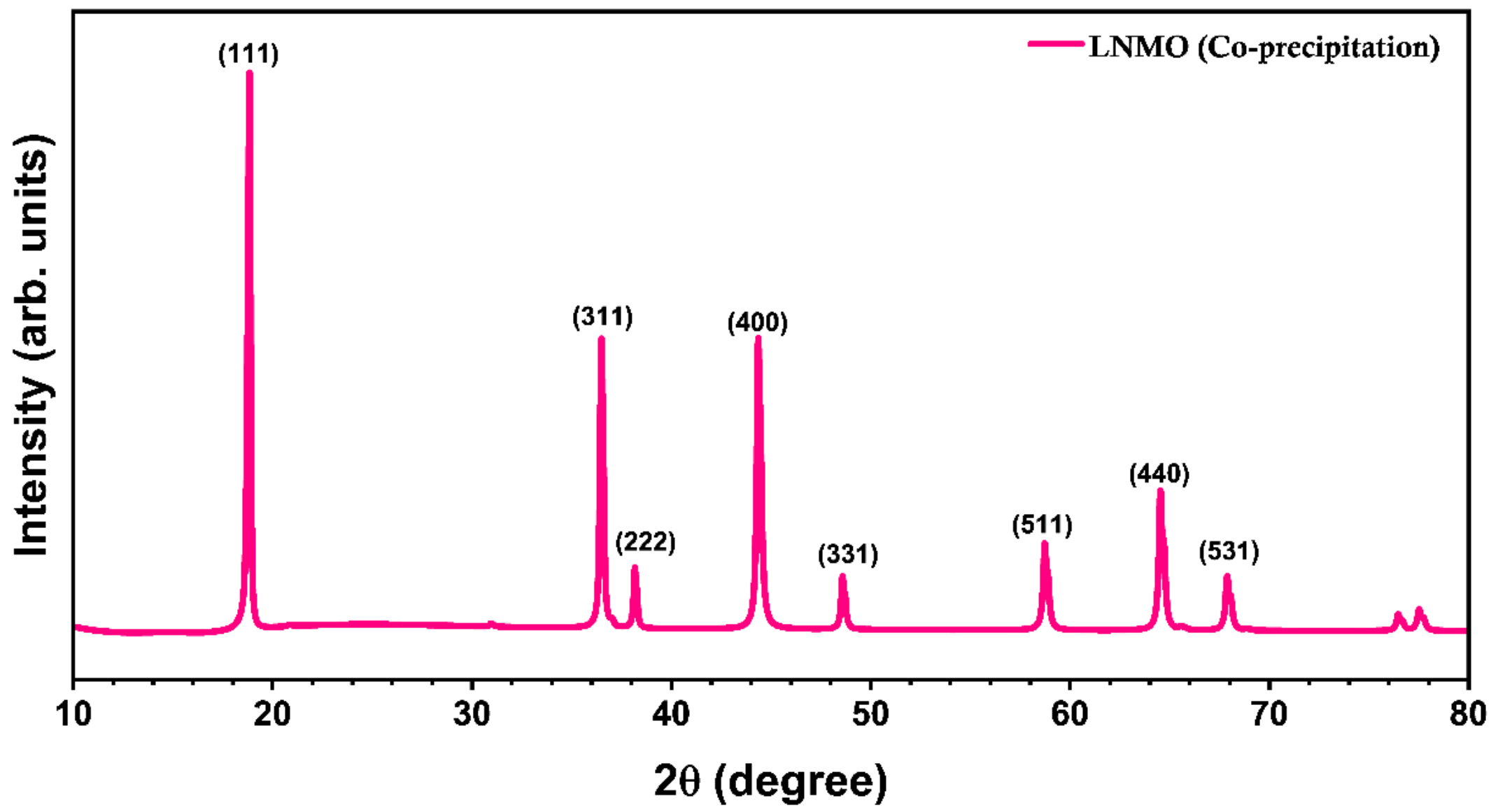

Figure S1. XRD pattern of co-precipitated LNMO

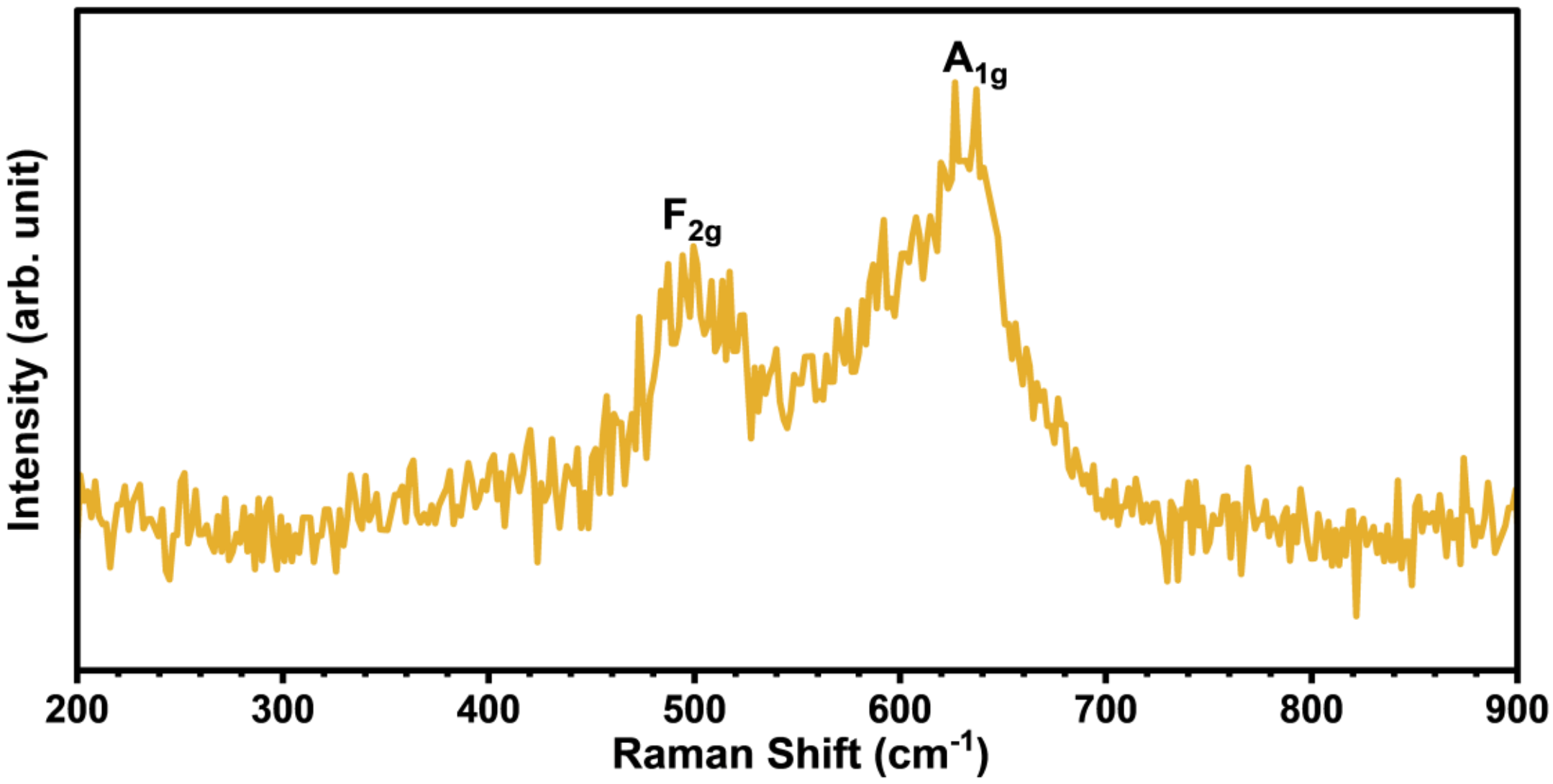

Figure S2: Raman spectra of LNMO

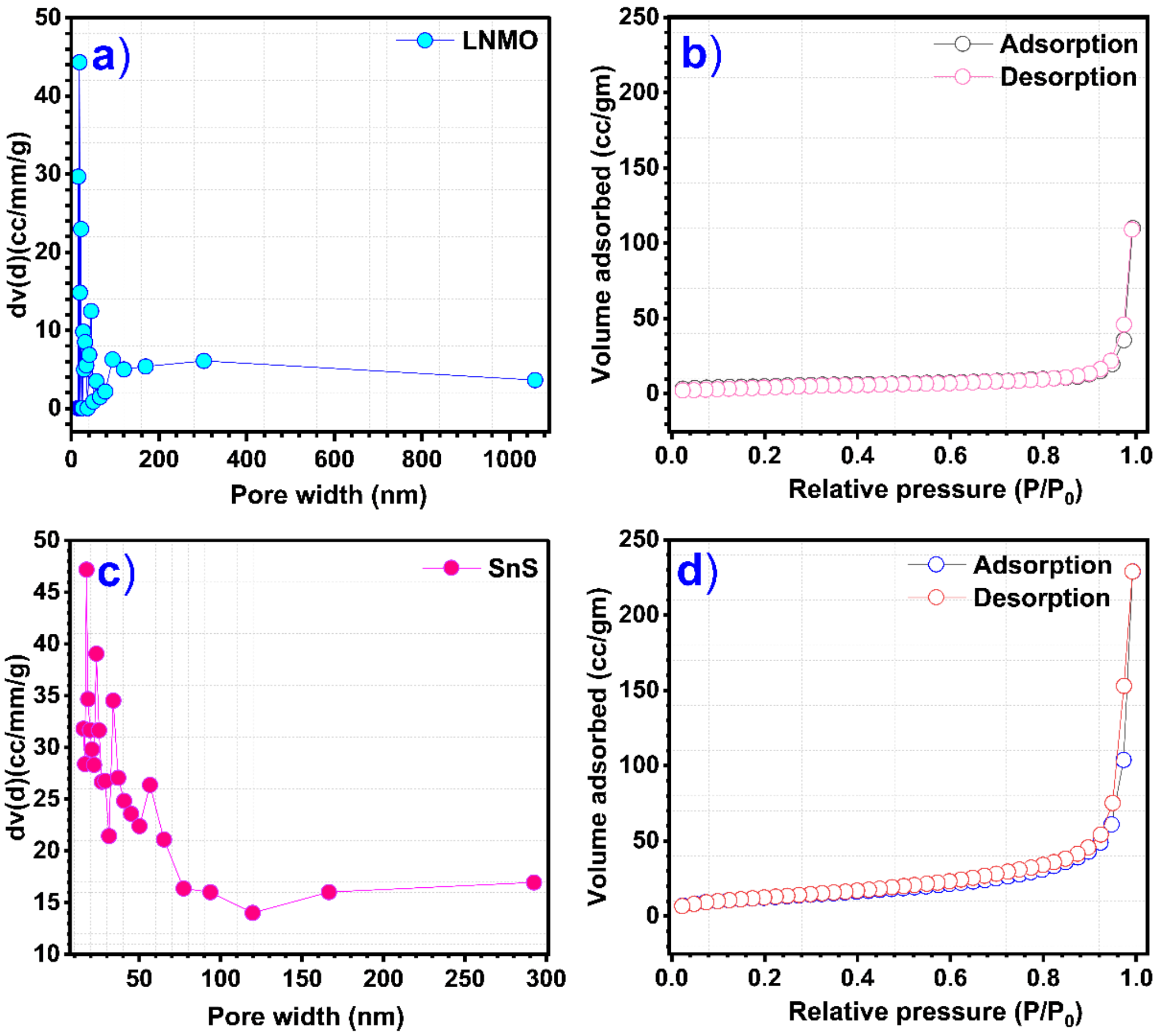

Figure S3. BJH pore size distribution of (a) E-spun LNMO, (c) SnS nanoparticles. Brunauer-Emmet-Teller (BET) nitrogen adsorption/desorption isotherm of (b) E-spun LNMO, and (d) SnS nanoparticles

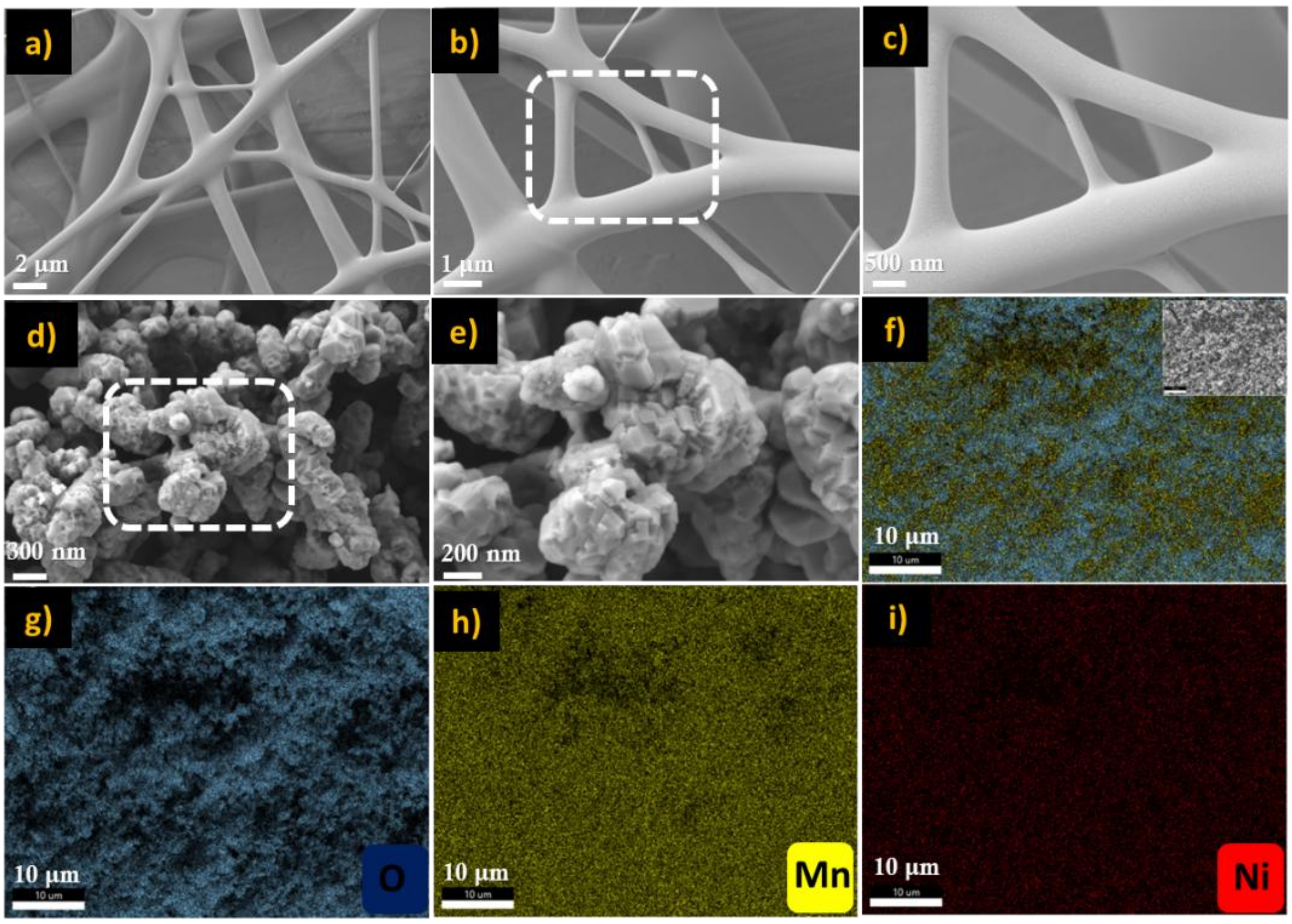

Figure S4. FE-SEM images at different magnifications: (a-c) LNM fibres, (d-e) co-precipitated LNMO, and (f-i) EDS elemental mapping of co-precipitated LNMO

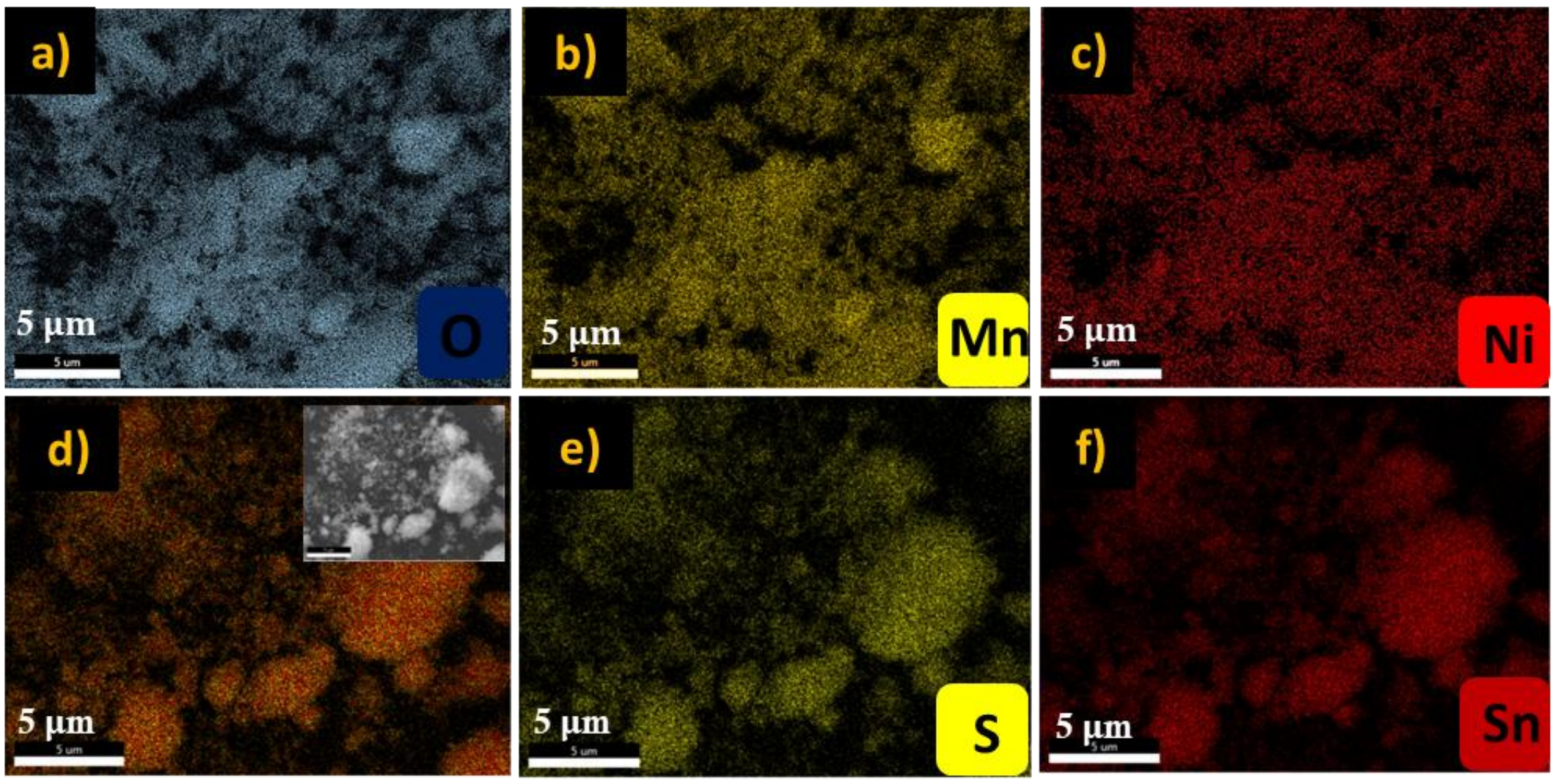

Fig S5. EDS elemental mapping of E-spun LNMO (a-c), and SnS (d-f)

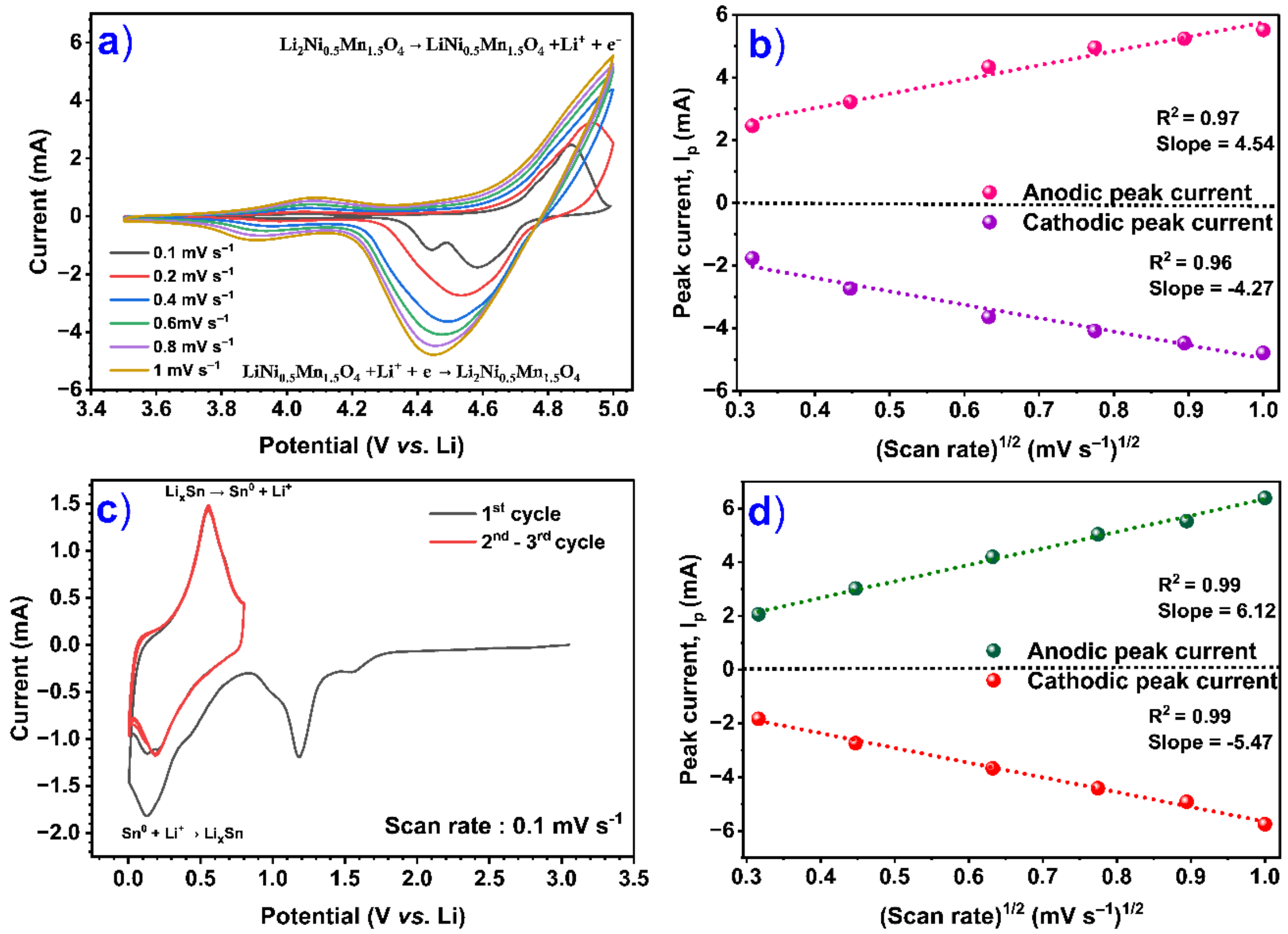

Figure S6. (a) CV of E-spun LNMO (high-voltage) at different scan rates (0.1 to 1 mV s^{-1}), (b & d) peak current (I_p) *vs.* square root of scan rate ($\nu^{1/2}$) plot for E-spun LNMO in the high and low-voltage region, respectively, and (c) CV of SnS at a scan rate of 0.1 mV s^{-1}

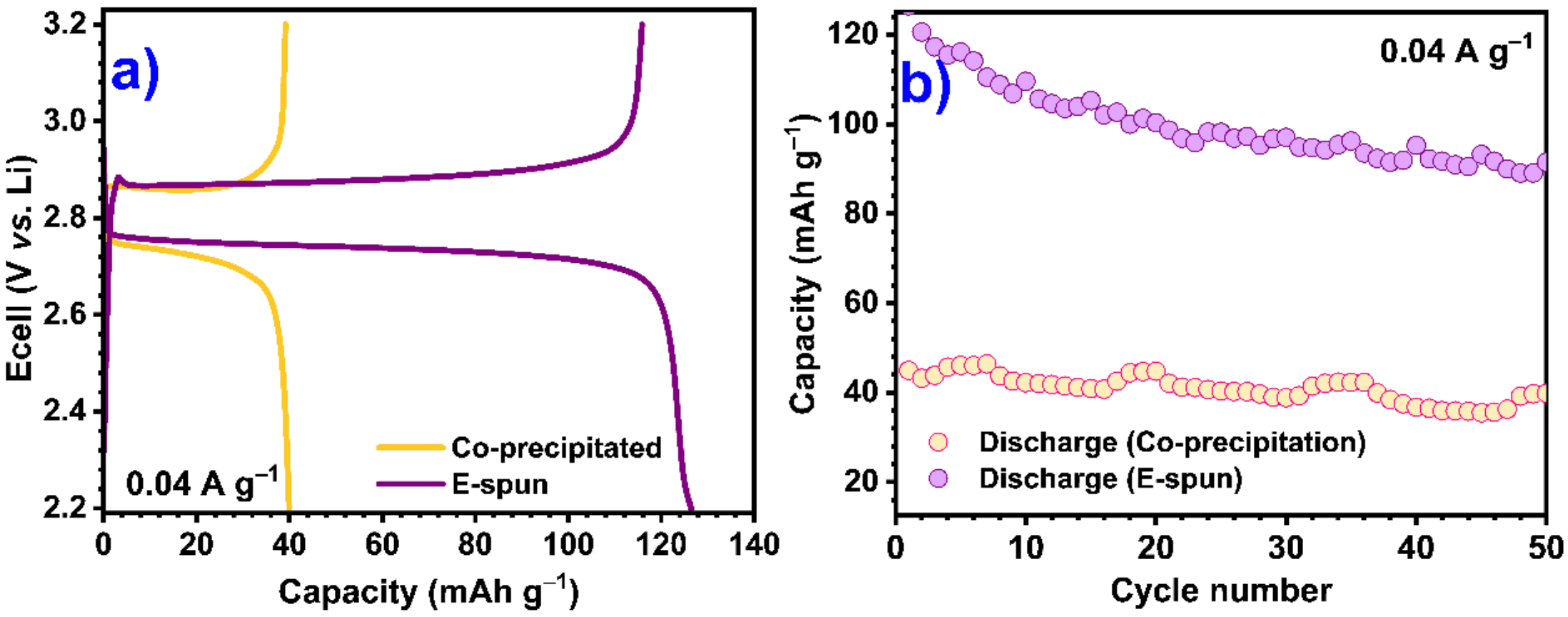

Figure S7. (a) Comparison of GCD curves of E-spun and co-precipitated LNMO at low voltage at 0.04 A g^{-1}, and (b) long-term cycling performance comparison of both at the same current density.

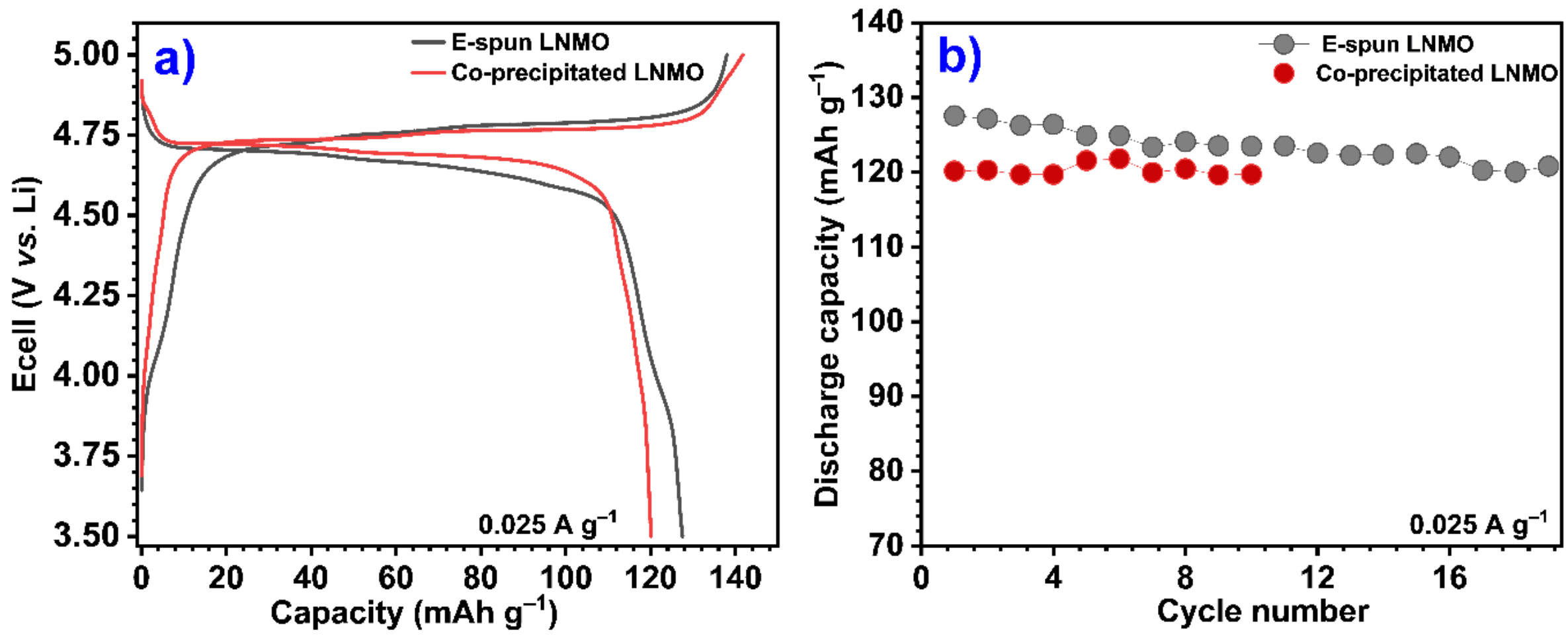

Figure S8. (a) Comparison of GCD curves of E-spun and co-precipitated LNMO at high voltage at 0.025 A g^{-1}, and (b) long-term cycling performance comparison of both at the same current density.

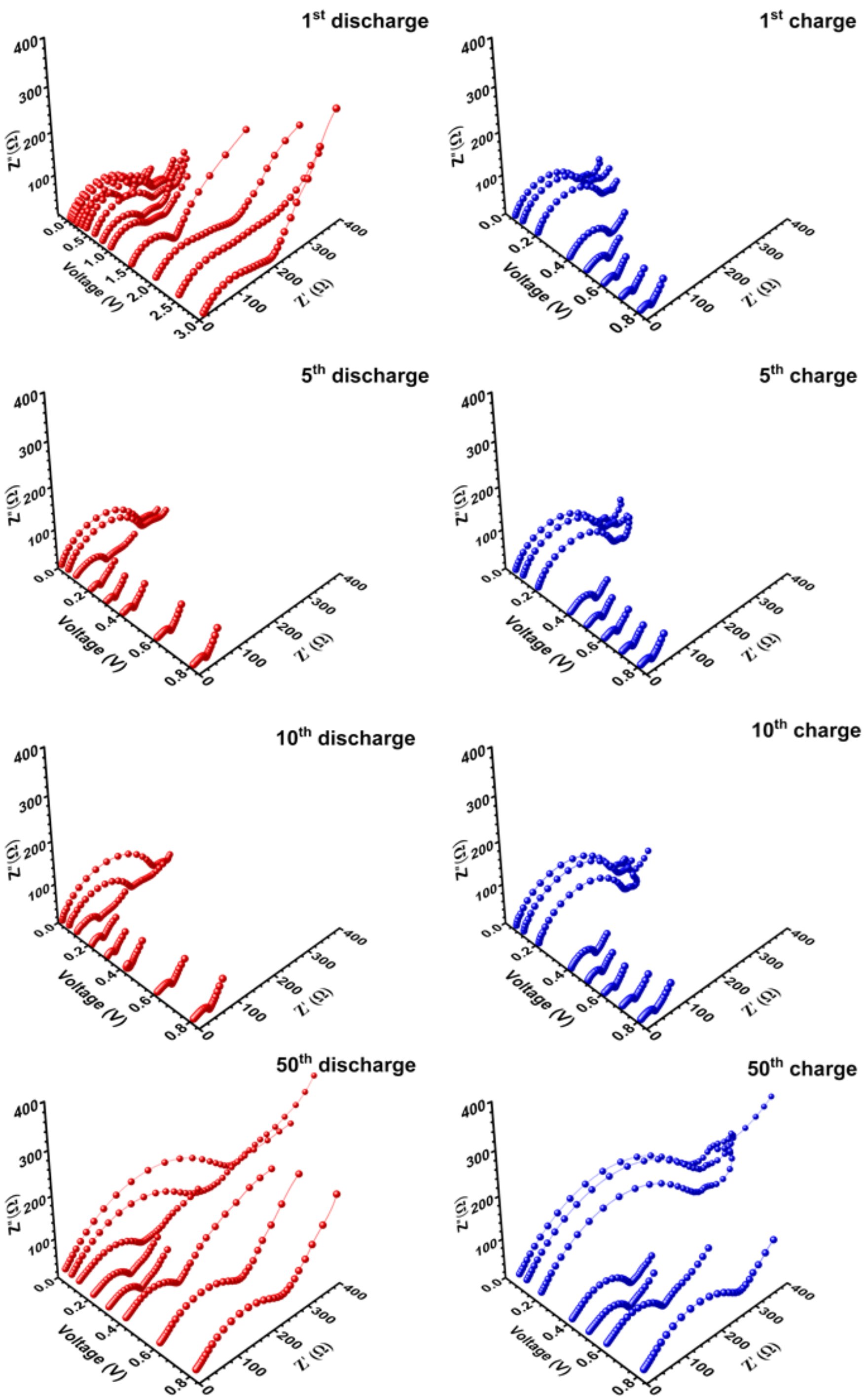

Figure S9. *In-situ* electrochemical impedance spectroscopy profile of Li/SnS half-cell with traces of 1st, 5th, 10th, and 50th cycles at different potentials.

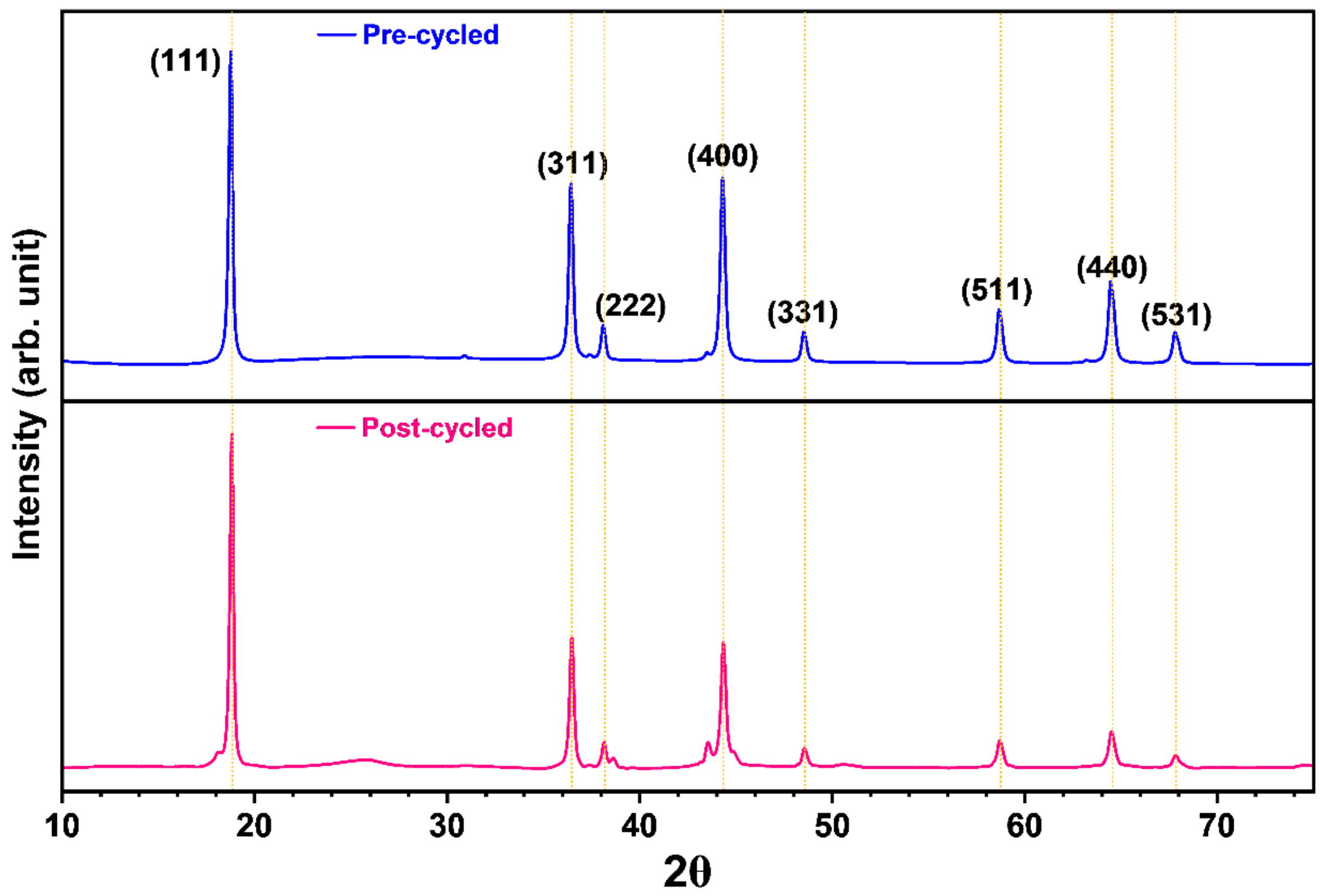

Figure S10: Comparison of the XRD pattern of LNMO before and after cycling.

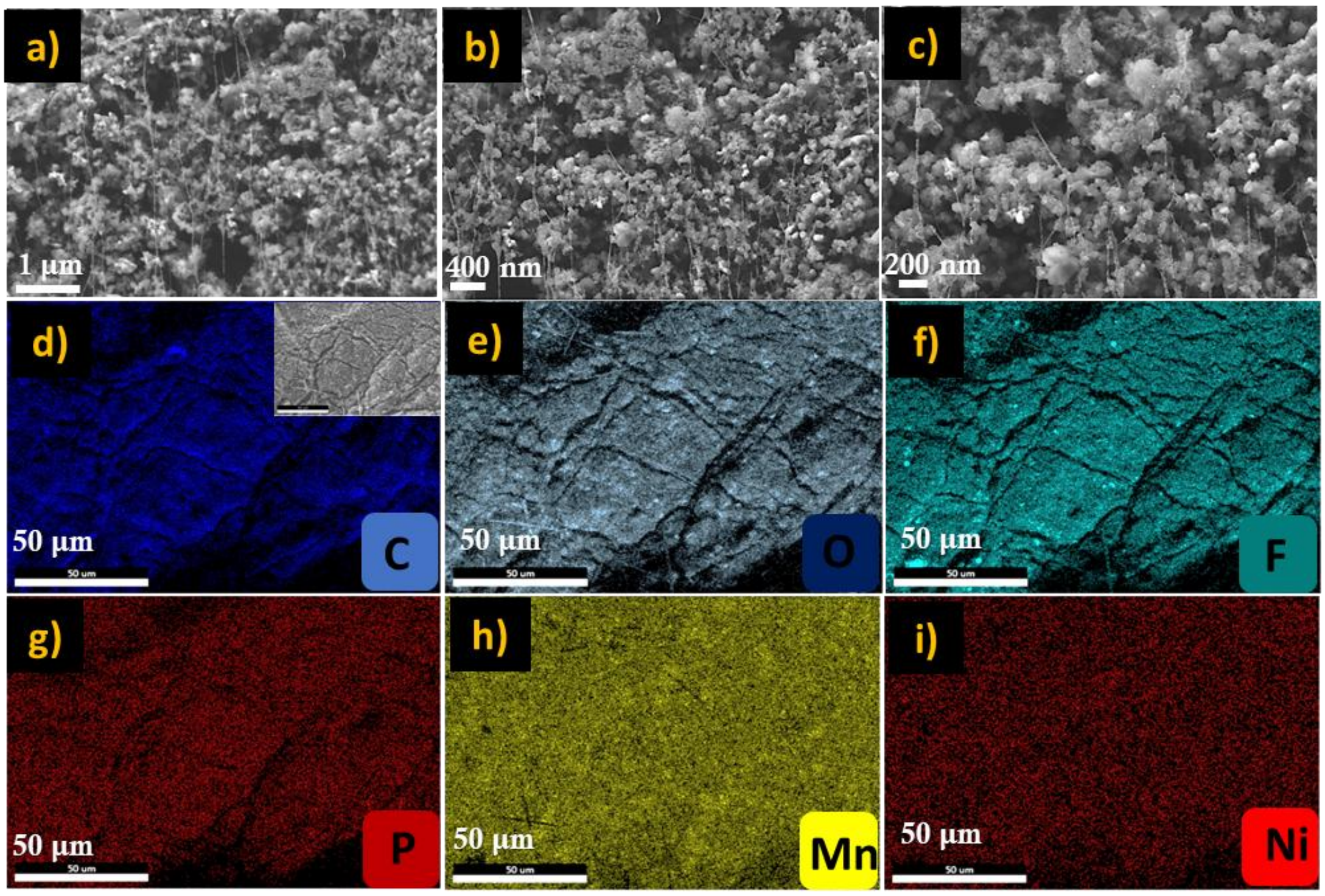

Figure S11. Post-mortem studies of LNMO: (a-c) FE-SEM images at different magnifications, and (d-i) elemental mapping.

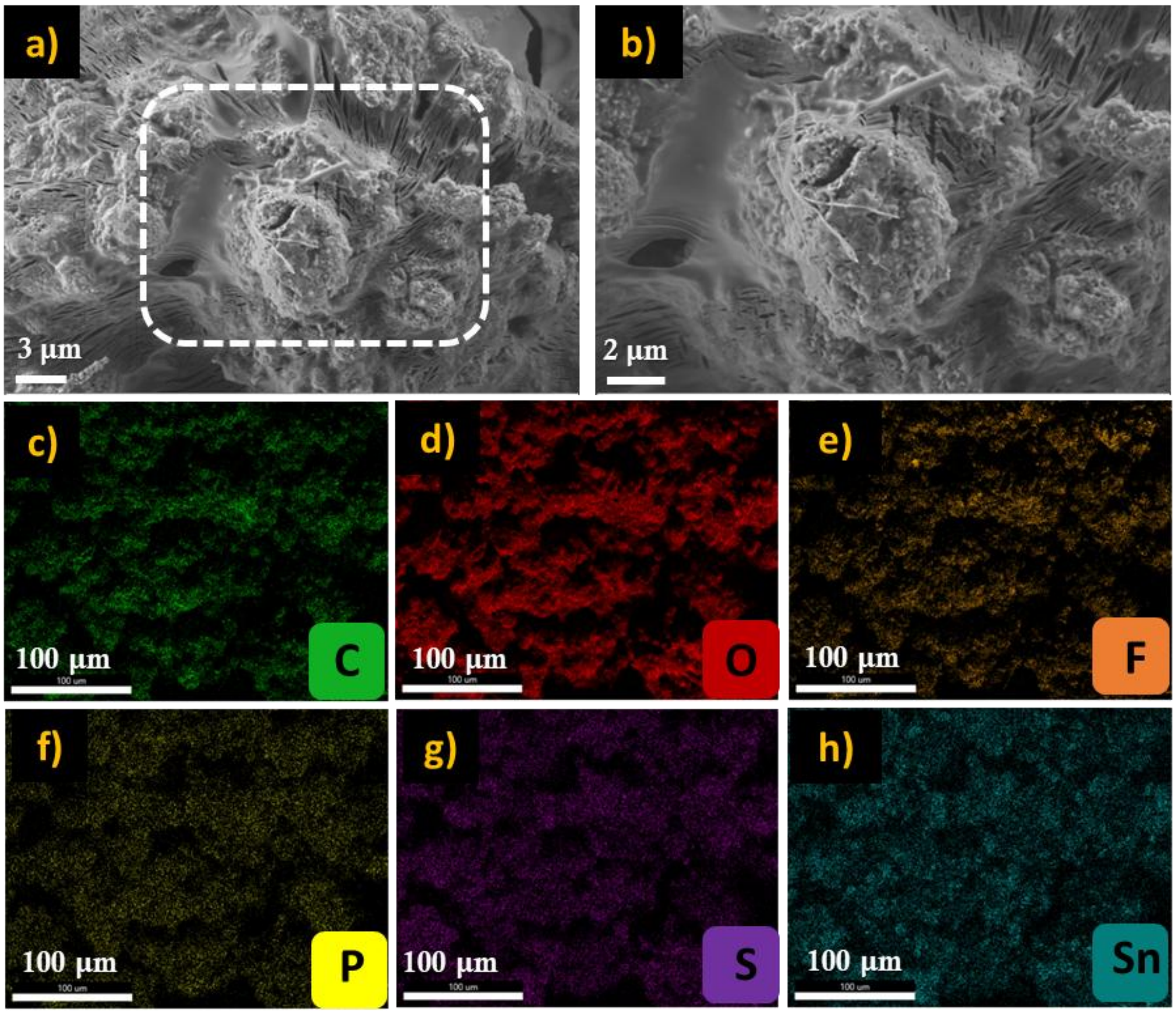

Figure S12. SnS post-mortem studies: (a-b) FE-SEM images, and (c-h) EDS elemental mapping.
